# Calcium-sensing receptor gene (*CASR*) polymorphisms and *CASR* transcript level concerning dyslipidemia in hemodialysis patients: a cross-sectional study

**DOI:** 10.1186/s12882-019-1619-0

**Published:** 2019-11-27

**Authors:** Alicja E. Grzegorzewska, Bartosz A. Frycz, Monika Świderska, Leszek Niepolski, Adrianna Mostowska, Paweł P. Jagodziński

**Affiliations:** 1 Department of Nephrology, Transplantology and Internal Diseases, Poznan University of Medical Sciences, Przybyszewskiego 49, 60-355 Poznań, Poland; 2 Department of Biochemistry and Molecular Biology, Poznan University of Medical Sciences, Święcickiego 6, 60-781 Poznań, Poland; 3 B.Braun Avitum Poland, Dialysis Center, Sienkiewicza 3, 64-300 Nowy Tomyśl, Poland

**Keywords:** *CASR*, Dyslipidemia, *ENHO*, Hemodialysis, *LXRA*, *RXRA*, Transcript leve

## Abstract

**Background:**

There is scarce data on *CASR* associations with dyslipidemia. We investigated in hemodialysis (HD) patients whether *CASR* single nucleotide polymorphisms (SNPs) rs7652589 and rs1801725 have associations with dyslipidemia and show epistatic interactions with SNPs of the energy homeostasis-associated gene (*ENHO)*, retinoid X receptor α gene (*RXRA*), and liver X receptor α gene (*LXRA*).

**Methods:**

The study included 1208 HD subjects. For diagnosis of dyslipidemia, both K/DOQI criteria and atherogenic index ≥3.8 were used. *CASR* rs1801725 was genotyped by TaqMan SNP Genotyping Assay, other SNPs – by high-resolution melting curve analysis or polymerase chain reaction-restriction fragment length polymorphism, as appropriate. Relative transcript levels of *CASR*, *ENHO*, *RXRA*, and *LXRA* were measured in peripheral blood mononuclear cells. The occurrence of dyslipidemic phenotypes concerning tested polymorphisms was compared using models of inheritance. Haplotypes were estimated using the Haploview 4.2 software. Epistatic interactions between tested SNPs were analyzed using the logistic regression and epistasis option in the PLINK software.

**Results:**

Rs7652589 indicated a greater probability of atherogenic dyslipidemia in the dominant inheritance model (OR 1.4, 95%CI 1.0–2.0, *P* = 0.026), principally because of increased triglyceride (TG) levels. The rs1801725 variant allele was associated with a decreased probability of dyslipidemia characterized by non-HDL-cholesterol ≥130 mg/dL and TG ≥200 mg/dL (OR 0.6, 0.4–0.9, *P* = 0.012). There were no epistatic interactions between *CASR* and *RXRA*, *LXRA*, and *ENHO* regarding dyslipidemia. Both rs7652589 and rs1801725 SNPs were not in linkage disequilibrium (D’ = 0.091, r^2^ = 0.003 for the entire HD group) and their haplotypes did not correlate with dyslipidemia. Relative *CASR* transcript was lower at a borderline significance level in patients harboring the rs1801725 variant allele compared with homozygotes of the major allele (0.20, 0.06–7.80 vs. 0.43, 0.04–5.06, *P* = 0.058). *CASR* transcript correlated positively with *RXRA* transcript (adjusted *P* = 0.001), *LXRA* transcript (adjusted *P* = 0.0009), *ENHO* transcript (borderline significance, adjusted *P* = 0.055), dry body weight (adjusted *P* = 0.035), and renal replacement therapy duration (adjusted *P* = 0.013).

**Conclusions:**

*CASR* polymorphisms (rs7652589, rs1801725) are associated with dyslipidemia in HD patients. *CASR* correlates with *RXRA*, *LXRA*, and *ENHO* at the transcript level. Further investigations may elucidate whether other *CASR* SNPs contribute to associations shown in this study.

## Background

Patients requiring hemodialysis (HD) treatment show multiple metabolic disturbances, including dyslipidemias. Lipid alterations, among them atherogenic dyslipidemia, contribute to initiation and progression of coronary artery disease (CAD), myocardial infarction (MI), and premature death. In end-stage renal disease patients, dyslipidemias were defined by the National Kidney Foundation/Kidney Disease Outcomes Quality Initiative (K/DOQI) as serum low-density lipoprotein (LDL)-cholesterol ≥100 mg/dL or simultaneously occurring non-high density lipoprotein (non-HDL)-cholesterol ≥130 mg/dL and triglycerides (TG) ≥ 200 mg/dL [1]. Atherogenic dyslipidemia is frequently referred to the atherogenic index calculated as TG to high-density lipoprotein (HDL)-cholesterol ratio which is equal to or over 3.8 [2].

Although 79.8% of HD patients are reported to have abnormal serum levels [1], the etiology of dyslipidemias in this group of patients is still insufficiently elucidated. In uremic subjects, atherosclerotic plaques and vascular calcifications are accompanied by mineral disorders, increased calcium-phosphorus product, and advanced secondary hyperparathyroidism [3].

Single nucleotide polymorphisms (SNPs) of calcium-sensing receptor gene (*CASR*) are predominantly associated with phenotypes of primary [4] and secondary hyperparathyroidism [5–8], and with idiopathic calcium nephrolithiasis [9, 10]. However, *CASR* expression is shown not only in parathyroid glands [11] and kidney tubules [10], but also in vascular smooth muscle cells [12], adipocytes and their progenitor cells [13, 14], human omental tissue [14], and hepatocytes [14, 15]. It is suggested that livers from obese patients may express higher levels of *CASR* transcripts [14]. Reduced *CASR* mRNA levels were attributed to variant alleles of *CASR* rs7652589 [7, 16] and rs1501899 [16]. An increase of Ca^2+^ in cytosol due to activation of the calcium-sensing receptor (CaSR) may influence adipogenesis and accumulation of TG in adipocytes [13]. It is suggested that CaSR has antilipolytic effect in human adipocytes [13]. On the other hand, CaSR activation by calcimimetic cinacalcet decreased adipocyte TG content by 20% [14]. Antilipolytic effect of calcimimetics was attributed to the allelic variant of *CASR* polymorphism rs1042636 (Arg990Gly) [17]. Additionally, up-regulation of CaSR was found to activate the peroxisome proliferator-activated receptor ɣ (PPARɣ) which is a transcription factor involved in the regulation of adipogenesis [18]. PPARs heterodimerize with retinoid X receptors (RXR) [19]. RXRα and liver X receptor α (LXRα) form the functional heterodimer LXRα-RXRα [20]. Up-regulation of LXRα diminishes the liver expression of the energy homeostasis-associated gene (*ENHO*) [21]. *ENHO*, *RXRA*, and *LXRA* SNPs were associated separately or jointly with dyslipidemia, MI, and survival in HD patients [22].

Several *CASR* SNPs were studied for their relationships with serum total cholesterol concentration, but results were negative, at least in renal transplant recipients [23]. The *CASR* R990G (rs1042636) polymorphism was associated with increased risk of hypertriglyceridemia in the Chinese population, especially in obese individuals, but other two nonsynonymous *CASR* coding region SNPs (A986S rs1801725, Q1011E rs1801726) were distributed similarly in hypertriglyceridemic and non-hypertriglyceridemic subjects [24]. In Caucasians, the variant allele in *CASR* rs1801725 (but not homozygosity of the variant allele) was reported to be an independent predictor of CAD, MI, and cardiovascular mortality [25].

As rs7652589 was previously associated with secondary hyperparathyroidism in HD patients [5–8] and rs1801725 was reported as related to CAD, MI, and cardiovascular mortality in Caucasians [25], we have chosen these two SNPs for searching their associations with dyslipidemia in HD subjects. Dyslipidemia may be a factor which together with calcium disturbances contributes to atherogenic cardiac disease and cardiovascular mortality. HD patients, presenting both dyslipidemia and secondary hyperparathyroidism, seem to be a unique group to study *CASR* polymorphisms that are associated with calcium disorders and possibly also with dyslipidemia. Both *CASR* SNPs (rs7652589 located 13 kbp upstream from the TATA box of promoter 1 and a missense variant rs1801725 located in exon 7 on chromosome 3) are not in linkage disequilibrium (LD), so their associations are not obvious and therefore worth to investigate.

The aim of our study was to investigate whether *CASR* SNPs (rs1801725, rs7652589) are associated with dyslipidemia in HD patients, or whether there is any interaction between *CASR* and other genes known as associated with lipogenesis, like *ENHO*, retinoid X receptor α gene (*RXRA*), or liver X receptor α gene (*LXRA*). *CASR*, *ENHO*, *RXRA*, and *LXRA* transcripts were also tested for correlations.

## Methods

### Patients

To be enrolled in the study, HD patients had to fulfill the following criteria:

1. not to show secondary causes of dyslipidemia (hypothyroidism, alcohol abuse, medication with anticonvulsants, corticosteroid therapy) and cachectic conditions causing decreases in serum lipids (neoplasms, enteropathies, liver cirrhosis),
2. not to receive treatment with cinacalcet at least for 6 months before determination of serum lipid profile,
3. to have a serum lipid profile determined in stable general condition.

HD patients were qualified as candidates for this study independently on treatment with lipid-lowering medications. However, to be included as not receiving lipid-lowering therapy, the patients had to be free from lipid-lowering medicines for at least 6 months before to determination of serum lipid profile used in this study. To be included as receiving lipid-lowering therapy, the patients had to undergo lipid-lowering medications for at least 6 months before the determination of serum lipid concentrations used in this study. Patients, who did not fulfill these criteria, were excluded.

HD subjects were diagnosed as having dyslipidemia by the use of the K/DOQI criteria [1] and also by applying the atherogenic index [2].

All the study participants (*n* = 1208) were Caucasians of Polish origin.

### Laboratory examinations

In all studied HD individuals, blood samples were taken before the midweek HD session for *CASR*, *ENHO*, *RXRA*, and *LXRA* polymorphisms, serum lipids (total cholesterol - TC, HDL-cholesterol, TG), and laboratory parameters routinely tested in HD subjects.

Serum lipids which were determined using enzymatic colorimetric tests (Roche Diagnostics, Mannheim, Germany). The LDL-cholesterol level was computed by the Friedewald equation [26]. If serum TG levels equal to or exceeding 400 mg/dL, LDL-cholesterol was determined directly (BioSystems S.A., Reagents and Instruments, Barcelona, Spain). Non-HDL cholesterol was calculated as the TC minus HDL-cholesterol.

### Genotyping

Tested SNPs in *CASR* (rs7652589, rs1801725), *ENHO* (rs2281997, rs72735260), *RXRA* (rs749759, rs10776909, rs10881578), and *LXRA* (rs2279238, rs7120118, rs11039155) were characterized using public databases including the NCBI dbSNP database (http://www.ncbi.nlm.nih.gov/ projects/SNP/) and the 1000 Genomes Browser (http://browser.1000genomes.org/ index.html). SNPs were selected based on variant (minor) allele frequency (MAF) exceeding 5% in the Caucasian population and gene LD patterns. Characteristics of the tested polymorphisms are displayed in Additional file 1: Table S1. Genomic locations of *CASR* rs7652589 (A < G) and rs1801725 (G > T) are shown in Additional file 1: Figure S1.

DNA for genotype analysis was extracted from blood lymphocytes by the salt-out extraction method. *CASR* rs7652589, *RXRA* SNPs, and *ENHO* SNPs were genotyped as previously described [7, 27]. Analysis of *CASR* rs1801725 variant was performed using predesigned TaqMan SNP Genotyping Assay according to the manufacturer’s instructions provided by Applied Biosystems (Applied Biosystems, Foster City, CA). Genotyping of *LXRA* SNPs performed using high-resolution melting curve (HRM) analysis on the Light Cycler 480 system (Roche Diagnostics, Germany). In brief, DNA fragments amplified with the use of specific primers were subjected to HRM with 0.1 °C increments in temperatures ranging from 70 to 92 °C. For quality control, approximately 20% of the randomly chosen samples were re-genotyped. Samples with ambiguous results were excluded from further statistical analyses.

Genotyping was performed using encoded blood samples.

*CASR* rs7652589 and *CASR* rs1801725 genotypes were obtained in 1139 and 1159 patients, respectively. *ENHO* rs2281997 was successfully genotyped in 1182 patients, *ENHO* rs72735260 – in 1183 subjects. Genotyping for *RXRA* SNPs was performed with success in 1196 patients for rs749759, in 1199 patients for rs10776909, and in 1200 for rs10881578. *LXRA* SNPs (rs2279238, rs7120118, rs11039155) were successfully genotyped in 1186, 1188, and 1189 patients, respectively.

Distributions of tested polymorphisms were in concordance with the Hardy-Weinberg equilibrium (HWE).

### Reverse transcription-quantitative polymerase chain reaction (qPCR) analysis

*CASR*, *ENHO*, *RXRA*, and *LXRA* transcripts were determined in 112 HD patients. Due to a risk of complications, mainly bleeding during the collection of tissue material in HD patients, the only available cell material for the transcript determination was that composed of peripheral blood mononuclear cells (PBMCs). They were isolated by density-gradient centrifugation using the Histopaque (Sigma-Aldrich, Missouri, United States). Cells were washed in phosphate-buffered saline, and total RNA isolation was performed according to the method of Chomczyński and Sacchi [28]. The concentration and integrity of the isolated RNA were assessed by spectrophotometric quantification and nondenaturing electrophoresis on a 2% agarose gel. RNA samples were treated with Ambion DNase I (Thermo Fisher Scientific, Inc., Waltham, MA, USA) and 1.5 μg of RNA was reverse-transcribed into complementary DNA (cDNA) using an M-MLV Reverse Transcriptase (Thermo Fisher Scientific, Inc., Waltham, MA, USA) according to the manufacturer’s protocol. Quantitative analyses were performed using a LightCycler® 480 Real-Time PCR system (Roche Diagnostics GmbH, Mannheim, Germany). The transcripts of target genes were quantified by the relative quantification method using a calibrator, as is described in the Relative Quantification Manual (Roche Diagnostics GmbH, Mannheim, Germany) [29]. The calibrator contained the cDNA mix from all analyzed samples. Each qPCR mix contained 1 μl of cDNA, 9 μl LightCycler 480 SYBR Green I Master Mix (Roche Diagnostics GmbH) and 0.25 μM of the corresponding primers. Primers sequence for *CASR* was used as previously [7] whereas all other primers were newly designed using OLIGO Primer Analysis Software (version 5.0; Molecular Biology Insights, Inc., Colorado Springs, CO, USA) in our laboratory. Sequences for these primers were used as follow: *ENHO*, F: CAGGCTCAACTCAGGCTCAG and R: GAGGAGGCTGTGCTGTCTGC; *LXRA*, F: CGAGGTGATGCTTCTGGAGA and R: CCTGGAGAACTCGAAGATGG; *RXRA* F: TCCTCTTTAACCCTGACTCC and R: AGAGCTTAGCGAACCTTCC. The transcript amounts were calculated as the ratio between the quantity of target transcript in a sample and target transcript in the calibrator. The portion of analyzed transcripts in each sample was standardized by transcripts of β_2_-microglobulin and hydroxymethylbilane synthase genes. In one patient, samples for *RXRA* and *LXRA* relative transcript amounts yielded ambiguous results and were excluded from statistical analyses.

### Statistical analysis

Percentages are shown for categorical variables. Continuous variables are expressed as medians and ranges due to their non-normal distribution according to the Shapiro–Wilk test. For comparison of continuous variables, the Mann–Whitney U test was applied. To compare dichotomous variables, Chi^2^ test, Chi^2^ test with Yates correction, Chi^2^ test for trend in proportions, and Fisher exact test were used, as appropriate.

For HWE analysis, the observed genotype frequencies were compared to the normal ones using the Chi^2^ test (*P* > 0.05 with df = 1 for balance). *P* values concerning associations of the tested SNPs with selected phenotypes were additionally evaluated using the BADGE system [30].

The occurrence of dyslipidemic phenotypes concerning tested polymorphisms was compared using models of inheritance (dominant, recessive, additive) in four HD groups:

1. composed of subjects dyslipidemic by K/DOQI criteria, atherogenic index, or both among patients not receiving lipid-lowering medication together with all patients treated with antilipemic medicines,
2. with an exclusion of patients in whom dyslipidemia was abolished by antilipemic medicines (patients who were dyslipidemic on antilipemic treatment were assumed to have the same type of dyslipidemia as before initiation of antilipemic medication),
3. comprised of patients not receiving antilipemic medicines,
4. composed of patients receiving antilipemic medication.

All four groups were compared with subjects free of dyslipidemia by both criteria not receiving antilipemic medication. Patients showing dyslipidemia by K/DOQI rules [1] or by the atherogenic index [2] were compared with patients without dyslipidemia by a respective criterion. Among patients dyslipidemic by K/DOQI criteria [1], the group showing plasma LDL-cholesterol concentration ≥ 100 mg/dL and the group presenting non-HDL-cholesterol ≥130 mg/dL and TG ≥ 200 mg/dL were also analyzed separately.

For selected categorical variables, Odds ratio (OR) and 95% confidence intervals (CIs) for OR were calculated. Chi-square test or Fisher’s test was used for statistical evaluation of OR. All probabilities were two-tailed, and *P*-value below 0.05 was considered significant.

To determine the associations of selected SNPs with appropriate phenotypes among other relevant patient characteristics, we used the primary logistic regression models with subsequent stepwise logistic regression and backward elimination for selection of significant variables among other possible determinants of tested dyslipidemic phenotypes.

Abovementioned statistical analyses were performed using R software version 3.4.0 [31], and Statistica version 12 (Stat Soft, Inc., Tulsa, Oklahoma, United States).

Pair-wise LD between tested SNPs was computed as both D’ and r^2^ using the genotype data from the tested sample and the Haploview 4.2 software (http://www.broad.mit.edu/mpg/haploview/).

Distribution of haplotypes was analyzed by the mentioned above Haploview 4.2 software. Haplotypes were statistically analyzed if their incidence in the examined group was over 1%. Statistical significance was evaluated using the 1000-fold permutation test.

The analysis of epistatic interactions between tested SNPs was performed by the logistic regression and epistasis option in the PLINK software http://zzz.bwh.harvard.edu/plink/. The false discovery rate (FDR) method was used to correct for multiple comparisons [32, 33].

## Results

### Patient characteristics

**Fig. 1:**
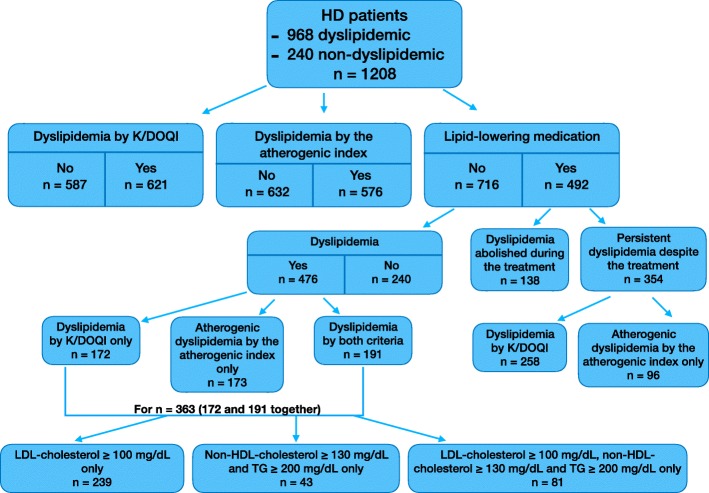
Serum lipid status in HD patients

Figure 1 presents the lipid status in the enrolled HD patients.

Lipid-lowering medications (statins, fibrates, or both) were used in 492 patients, of whom 138 (28.0% of total treated) abolished dyslipidemia by both criteria. HD patients diagnosed as dyslipidemic by K/DOQI criteria [1] (*n* = 621) did not differ in frequency of lipid-lowering medication compared with subjects without dyslipidemia by the same criteria (*n* = 587). HD patients showing the atherogenic index ≥3.8 and receiving lipid-lowering medication revealed the TG/HDL-cholesterol ratio of 6.1 (3.8–49.7) (*n* = 272), whereas HD subjects showing the atherogenic index ≥3.8 and not treated had the TG/HDL-cholesterol ratio of 5.8 (3.8–34.5) (*n* = 304; *P*-value 0.047).

Additional file 1: Table S2 shows data of HD patients stratified by serum lipid status. Characteristics of patients dyslipidemic by K/DOQI or the atherogenic index were compared with those of non-dyslipidemic subjects by the respective criterion and with those of non-dyslipidemic patients by both criteria not receiving lipid-lowering medication (Additional file 1: Table S3). Compared to non-dyslipidemic subjects, HD patients dyslipidemic by K/DOQI criteria were the most frequent women, showed higher body mass index (BMI), and lower serum total alkaline phosphatase (ALP) activity. Subjects with atherogenic dyslipidemia revealed more frequently CAD and higher BMI (Additional file 1: Table S2 and Additional file 1: Table S3).

### *CASR* SNPs and dyslipidemia

Additional file 1: Table S4 and Additional file 1: Table S5 show statistical analyses of associations between tested *CASR* SNPs and dyslipidemia. Relationships at *P*-value < 0.05 for comparisons of the examined dyslipidemic group with a group without dyslipidemia by a respective criterion and also with a non-dyslipidemic group without antilipemic medication were taken for further analyses. Such associations were shown only in the group not receiving lipid-lowering medications. By the Better Associations for Disease and GEnes (BADGE) system [30], there was the fifth-class association between *CASR* rs7652589 and dyslipidemia diagnosed by the atherogenic index in the dominant model of inheritance. Carriers of the variant allele showed about 1.5-fold higher risk of dyslipidemia diagnosed by the atherogenic index compared with homozygotes of the major allele (Table 1). Prevalence of CAD was not associated with *CASR* rs7652589 in this group (Additional file 1: Table S6).

**Table 1:** *CASR* rs7652589 and dyslipidemia diagnosed by atherogenic index in HD patients not receiving lipid-lowering medication

Genotypes, MAF, HWE	Patients with atherogenic dyslipidemia	Patients without atherogenic dyslipidemia	Comparison of patients with atherogenic dyslipidemia and without atherogenic dyslipidemia Odds ratio (95% CI), *P*-value^1^	Patients without any dyslipidemia	Comparison of patients with atherogenic dyslipidemia and without any dyslipidemia Odds ratio (95% CI), *P*-value^1^
P_*trend*_^2^ = 0.095, P_*genotype*_^1^ = 0.072				P_*trend*_^2^ = 0.169, P_*genotype*_^1^ = **0.038**	
GG	91 (32.6)	159 (41.1)	**Reference**	93 (42.5)	**Reference**
AG	147 (52.7)	173 (44.7)	1.485 (1.058–2.083), **0.022**	91 (41.6)	1.651 (1.118–2.438), **0.011**
AA	41 (14.7)	55 (14.2)	1.302 (0.806–2.104), 0.279	35 (16.0)	1.197 (0.701–2.046), 0.510
AA + AG vs GG	188 (67.4)	194 (58.9)	1.441 (1.044–1.988), **0.026**	126 (57.5)	1.525 (1.057–2.200), **0.024**
AA vs GG + AG	41 (14.7)	55 (14.2)	1.040 (0.672–1.610), 0.861	35 (16.0)	0.906 (0.555–1.479), 0.692
MAF	(0.41)	(0.37)	1.208 (0.966–1.510), 0.098	(0.37)	1.198 (0.926–1.549), 0.169
*P*-value for HWE	0.138	0.475		0.116	

Significant differences are indicated using a bold font.

1 – Pearson’s chi-squared test; 2 – Cochran-Armitage trend test

HD patients harboring the variant allele of *CASR* rs1801725 revealed about the 1.7-fold lower risk of dyslipidemia diagnosed by non-HDL-cholesterol ≥130 mg/dL and TG ≥ 200 mg/dL compared with homozygotes of the major allele (Table 2). Subjects showing homozygosity of the variant allele of *CASR* rs1801725 revealed 6.8-fold lower prevalence of CAD than those with homozygosity of the major allele as well as the 6.5-fold lower frequency of CAD than those being the major homozygotes and heterozygotes of *CASR* rs1801725 (Additional file 1: Table S7). All these associations represented the fifth-class by the BADGE system [30].

**Table 2:** *CASR* rs1801725 and dyslipidemia in patients not receiving lipid-lowering medication

Genotypes, MAF, HWE	Patients with dyslipidemia diagnosed with non-HDL-cholesterol ≥ 130 mg/dL and TG ≥ 200 mg/dL	Patients without dyslipidemia diagnosed with non-HDL-cholesterol ≥ 130 mg/dL and TG ≥ 200 mg/dL	Comparison of patients with dyslipidemia diagnosed with non-HDL-cholesterol ≥ 130 mg/dL and TG ≥ 200 mg/dL and without dyslipidemia of this type Odds ratio (95% CI), *P*-value^1^	Patients without any dyslipidemia	Comparison of patients with dyslipidemia diagnosed with non-HDL-cholesterol ≥ 130 mg/dL and TG ≥ 200 mg/dL and without any dyslipidemia Odds ratio (95% CI), *P*-value^1^
P_*trend*_^2^ = **0.010**, _P*genotype*_^1,3^ = **0.026**				P_*trend*_^2^ = **0.023**, _P*genotype*_^1,3^ = 0.060	
GG	92 (78.6)	386 (67.8)	**Reference**	159 (68.5)	**Reference**
GT	25 (21.4)	169 (29.7)	0.621 (0.385–1.001), **0.049**	67 (28.9)	0.645 (0.381–1.091), 0.101
TT	0 (0)	14 (2.5)	0.144 (0.009–2.437), 0.083^3^	6 (2.6)	0.133 (0.007–2.381), 0.091^3^
TT + GT vs GG	25 (21.4)	183 (32.2)	0.573 (0.356–0.922), **0.021**	73 (31.5)	0.592 (0.351–0.997), **0.048**
TT vs GG + GT	0 (0)	14 (2.5)	0.163 (0.010–2.752), 0.144^3^	6 (2.6)	0.148 (0.008–2.655), 0.185^3^
MAF	(0.11)	(0.17)	0.571 (0.367–0.889), **0.012**	(0.17)	0.583 (0.361–0.942), **0.026**
*P*-value for HWE	0.196	0.372		0.736	

Significant differences are indicated using a bold font.

1 – Pearson’s chi-squared test; 2 – Cochran-Armitage trend test, 3 – Fisher’s test

### Haplotype frequencies and epistatic interactions

*CASR* haplotypes and epistatic interactions between *CASR*, *ENHO*, *RXRA*, and *LXRA* were analyzed in patients not receiving lipid-lowering medication. Dyslipidemic subjects were tested against patients without dyslipidemia by the respective criterion or against non-dyslipidemic subjects not receiving lipid-lowering medicines.

Both rs7652589 and rs1801725 SNPs did not show LD: D’ = 0.091, r^2^ = 0.003 for the entire HD group (Additional file 1: Figure S1). *CASR* haplotypes were not associated with dyslipidemia (Additional file 1: Table S8).

Epistatic interactions between tested SNPs, significant in unadjusted analyses, are shown in Table 3. Like other authors [34], we have indicated interactions with FDR ≤0.25. *CASR* SNPs did not show gene-gene interactions with other tested genes concerning types of dyslipidemia if FDR criterion ≤0.25 was not introduced. Epistatic interaction between *ENHO* rs2281997 and *RXRA* rs749759 resulted in about 1.9-fold higher frequency of dyslipidemia diagnosed by LDL-cholesterol ≥100 mg/dL, whereas the interaction between *RXRA* rs10881578 and *RXRA* rs749759 correlated with the approximately 2.4-fold lower occurrence of dyslipidemia diagnosed by non-HDL-cholesterol ≥130 mg/dL and TG ≥ 200 mg/dL.

**Table 3:** Interactions between tested SNPs significant in unadjusted analyzes in HD patients not receiving lipid-lowering medication

CHR1	GENE1	SNP1	CHR2	GENE2	SNP2	The odds ratio for interaction	Chi-square	*P*-value	FDR-adjusted *P*-value
Patients with dyslipidemia by K/DOQI vs. patients without this phenotype									
9	*ENHO*	rs2281997	9	*RXRA*	*RXRA*	1.7690	7.007	0.0081	0.3645
Patients with LDL-cholesterol ≥100 mg/dL vs. patients without this phenotype									
3	*CASR*	rs1801725	9	*RXRA*	rs10881578	0.6030	4.048	0.0442	0.9916
9	*ENHO*	rs2281997	9	*RXRA*	rs749759	1.8920	8.794	0.0030	**0.1350**
Patients with non-HDL-cholesterol ≥130 mg/dL and TG ≥ 200 mg/dL vs patients without this phenotype									
9	*ENHO*	rs2281997	11	*LXRA*	rs2279238	2.4220	5.962	0.0146	0.3285
9	*RXRA*	rs10881578	9	*RXRA*	rs749759	0.4213	9.829	0.0017	**0.0765**
Patients with atherogenic index ≥3.8 vs. patients without this phenotype									
3	*CASR*	rs7652589	9	*ENHO*	rs72735260	0.5744	4.680	0.0305	0.8595
9	*ENHO*	rs2281997	11	*LXRA*	rs2279238	1.7750	4.298	0.0382	0.8595
Patients with dyslipidemia by K/DOQI vs. patients without dyslipidemia by all used criteria									
9	*ENHO*	rs2281997	9	*RXRA*	rs749759	1.767	5.528	0.0187	0.8415
Patients with LDL-cholesterol ≥100 mg/dL vs. patients without dyslipidemia by all used criteria									
9	*ENHO*	rs2281997	9	*RXRA*	rs749759	1.945	6.839	0.0089	0.4005
Patients with non-HDL-cholesterol ≥130 mg/dL and TG ≥ 200 mg/dL vs. patients without dyslipidemia by all used criteria									
9	*ENHO*	rs2281997	11	*LXRA*	rs2279238	2.604	5.017	0.0251	0.5648
9	*RXRA*	rs10881578	9	*RXRA*	rs749759	0.485	5.743	0.0166	0.5648
Patients with atherogenic index ≥3.8 vs. patients without dyslipidemia by all used criteria									
3	*CASR*	rs7652589	9	*ENHO*	rs72735260	0.564	3.904	0.0482	0.8162

*P*-values adjusted for FDR equal to or below 0.25 are considered significant and are indicated using a bold font.

*Abbreviations*: *CHR1* Chromosome of first SNP, *SNP1* Identifier for first SNP, *GENE1* Gene of the first SNP, *CHR2* Chromosome of second SNP, *SNP2* Identifier for second SNP, *GENE2* Gene of the second SNP, *P*-value 1df asymptotic *P*-value, FDR-adjusted *P*-value *P*-value adjusted for false discovery rate

### *CASR* SNPs and serum lipids

In HD patients not receiving lipid-lowering medication (*n* = 716), there were 172 (24.0% of total) subjects showing dyslipidemia by K/DOQI, 113 (15.8% of total) with dyslipidemia by atherogenic index, and 191 (26.7% of total) showing dyslipidemia by both criteria. Non-dyslipidemic patients (*n* = 240) comprised of 33.5% of total. LDL-cholesterol correlated with non-HDL cholesterol dependently on serum TG levels: Spearman’s rank-order correlation coefficient was 0.962 at TG < 150 mg/dL, 0.953 at ≥150–250 mg/dL, and 0.816 at > 250 mg/dL (Additional file 1: Figure S2).

Table 4 shows the associations of *CASR* SNPs with serum lipids in HD patients not receiving lipid-lowering medication. The atherogenic index (the TG/HDL-cholesterol ratio) as a continuous variable correlated with *CASR* rs7652589 in the dominant and additive models of inheritance. Increased serum TG levels predominantly participated in the higher atherogenic index in homozygotes for the variant rs7652589 allele. Such an analysis for rs1801725 did not reveal significant associations with serum lipids (Additional file 1: Table S9).

**Table 4:** *CASR* rs7652589 polymorphic variants and serum lipids in HD patients not receiving lipid-lowering medication (*n* = 666)

Parameter	GG	AG	AA	Model of inheritance	*P*-value^1^	*P*-value^2^
	*n* = 250	*n* = 320	*n* = 96			
Total cholesterol, mg/dL	171.5 (72–282)	174.5 (65–363)	164.5 (92–296)	AG + AA vs. GG	0.883	**0.024**
				AA vs. GG + AG	0.311	0.420
				AA vs. GG	0.442	0.097
HDL-cholesterol, mg/dL	42 (6–94)	39 (10–118)	39 (17.3–82)	AG + AA vs GG	**0.011**	0.864
				AA vs. GG + AG	0.376	0.455
				AA vs. GG	0.070	0.412
Triglycerides, mg/dL	131.3 (40–585)	140.5 (35–1105)	140 (35–406)	AG + AA vs. GG	0.067	**0.027**
				AA vs. GG + AG	0.680	0.284
				AA vs. GG	0.671	0.061
LDL-cholesterol, mg/dL	94.6 (27.8–208.4)	98 (20–350)	92.5 (27–369)	AG + AA vs. GG	0.705	0.126
				AA vs. GG + AG	0.480	0.545
				AA vs. GG	0.612	0.239
Non-HDL-cholesterol, mg/dL	125 (52–234)	132 (8–282)	120 (58–262)	AG + AA vs. GG	0.430	**0.037**
				AA vs. GG + AG	0.432	0.332
				AA vs. GG	0.737	0.079
TG/HDL-cholesterol ratio	3.1 (0.6–30.8)	3.6 (0.4–34.5)	3.4 (0.5–15)	AG + AA vs. GG	**0.015**	**0.037**
				AA vs. GG + AG	0.971	0.177
				AA vs. GG	0.325	**0.041**

Conversion factors to SI units are as follows: for cholesterols – 1 mg/dL = 0.0259 mmol/L, for triglycerides – 1 mg/dL = 0.0113 mmol/L.

Significant differences are indicated using a bold font.

1 – Mann Whitney test; 2 – *P*-value for rs7652589 SNP in a linear regression model including gender, age, BMI, diabetic nephropathy, coronary artery disease, and alkaline phosphatase activity

### *CASR* SNPs as correlates of dyslipidemia and CAD among other variables

We have analyzed whether *CASR* SNPs shown as associated with serum lipid status and CAD remained relevant also among parameters significantly differing HD subjects as shown in Additional file 1: Table S3 [gender, age, diabetic nephropathy, renal replacement therapy (RRT) duration, CAD, BMI, and ALP activity]. For CAD, we have also included dyslipidemia diagnosed by non-HDL-cholesterol ≥130 mg/dL and TG ≥ 200 mg/dL [1] as a possible explanatory variable. We performed all analyses in patients not receiving lipid-lowering medication.

Variables significantly associated with dyslipidemia diagnosed by atherogenic index [2] included BMI (OR 1.13, 95%CI 1.07–1.19, *P* = 0. 2.0E-6), RRT duration (OR 1.06, 95%CI 1.02–1.10, *P* = 0.007), and *CASR* rs7652589 in dominant model of inheritance (OR 1.58, 95%CI 1.00–2.49, *P* = 0.048). The atherogenic index expressed as a continuous variable correlated independently with *CASR* rs7652589 in dominant model of inheritance (β = 0.10 ± 0.05, *P* = 0.021) together with BMI (β = 0.26 ± 0.05, *P* = 1.1E-8) and diabetic nephropathy (β = 0.09 ± 0.05, *P* = 0.044). However, an association of rs7652589 with serum TG concentrations was borderline (β = 0.08 ± 0.05, *P* = 0.083) among other tested variables of which only BMI correlated significantly with TG (β = 0.25 ± 0.05, *P* = 5.2E-8).

Dyslipidemia diagnosed by non-HDL-cholesterol ≥130 mg/dL and TG ≥ 200 mg/dL [2] showed an association with BMI (OR 1.12, 95%CI 1.06–1.19, *P* = 1.7E-4), male gender (OR 0.41, 95%CI 0.24–0.72, *P* = 0.002), and *CASR* rs1801725 in dominant model of inheritance (OR 0.52, 95%CI 0.28–0.98, *P* = 0.042). *CASR* rs1801725 did not reveal an independent correlation with CAD (OR 0.26, 95%CI 0.03–2.34, *P* = 0.231). Age (OR 1.04, 95%CI 1.02–1.06, *P* = 1.2E-4), diabetic nephropathy (OR 2.02, 95%CI 1.11–3.69, P = 0.021), and BMI (OR 1.07, 95%CI 1.01–1.14, *P* = 0.024) were significant explanatory variables for CAD among HD patients not receiving lipid-lowering medication.

### *CASR* SNPs and lipid-lowering treatment

There were no differences in the distribution of *CASR* SNPs in HD subjects who showed specific types of dyslipidemia despite lipid-lowering medication and those who did not demonstrate this kind of dyslipidemia or were non-dyslipidemic without lipid-lowering therapy (Additional file 1: Table S4 and Additional file 1: Table S5).

### Correlations of *CASR*, *ENHO*, *RXRA*, and *LXRA* transcripts

In unadjusted analyses, transcripts of tested genes correlated significantly with RRT duration (*CASR*, *ENHO*), dry body weight (*RXRA*, *LXRA*), BMI (*RXRA*, *LXRA*), and serum albumin concentration (*LXRA*, *ENHO*) (Additional file 1: Table S10). If all tested variables were adjusted concerning RRT duration, dry body weight, and albumin level, as appropriate, *CASR* transcript correlated positively and *ENHO* transcript inversely with RRT duration. Patients on RRT > 5 years (*n* = 53) showed relative *CASR* transcript level of 0.470 (0.056–7.796), whereas subjects on RRT < 1 year (*n* = 20) had *CASR* transcript of 0.181 (0.041–3.702) (*P* = 0.010, Mann-Whitney test). Higher body mass correlated positively with *RXRA* and *CASR* transcript levels, and negatively – with *LXRA* transcript amounts. Serum albumin positively correlated with *ENHO* transcript. *LXRA* transcript was positively associated with CAD at the borderline level of significance. Tested transcripts did not correlate with gender, age, diabetic nephropathy, types of dyslipidemia, active HBV/HCV infection, liver enzyme activities but ALP with *RXRA* transcript, inflammatory state assessed by plasma C-reactive protein, or lipid-modifying treatment (Additional file 1: Table S11).

In univariate analysis, *CASR* transcript was lower at a borderline significance level in patients harboring the rs1801725 variant allele compared with homozygotes of the major allele (0.20, 0.06–7.80 vs. 0.43, 0.04–5.06, *P* = 0.058). For *RXRA* rs10776909, associations between polymorphic variants and transcript levels were borderline in the additive and recessive inheritance models in univariate analysis and also after adjustment for RRT duration, dry body weight, and albumin level. Homozygosity of the variant allele in *LXRA* rs7120118 was associated with lower transcript levels, significantly in the recessive model of inheritance in univariate analysis and also after adjustment for RRT duration, dry body weight, and albumin level (Additional file 1: Table S12).

Relative levels of transcripts correlated with one another. *CASR* transcript positively correlated with *RXRA*, *LXRA*, and *ENHO* transcripts in patients not receiving lipid-lowering medication (Table 5).

**Table 5:** Relative *CASR*, *RXRA*, *LXRA*, and *ENHO* transcript amounts and their correlations in HD patients

Gene	Relative transcript amount Median (min-max)	A *p*-value for a univariate regression model, *P*-value for a multivariate regression model including RRT duration, dry body mass, and serum albumin concentration		
		*RXRA*	*LXRA*	*ENHO*
All tested HD patients (*n* = 112)				
*CASR*	0.320 (0.041–7.796)	**0.001; 0.001**	**0.015; 0.0009**	0.087; 0.055
*RXRA*	0.850 (0.095–2.813)		**0.018; 1.4E-5**	**2.2E-9; 2.6E-8**
*LXRA*	1.014 (0.012–7.117)			0.688; 0.246
*ENHO*	0.666 (0.030–2.524)			
Patients not receiving lipid-lowering treatment (*n* = 77)				
*CASR*	0.315 (0.041–7.796)	**0.002; 0.003**	**0.040; 0.003**	0.083; **0.044**
*RXRA*	0.875 (0.095–2.813)		**0.029; 2.2E-5**	**7.8E-6; 2.8E-5**
*LXRA*	0.968 (0.012–7.117)			0.902; 0.257
*ENHO*	0.752 (0.030–2.466)			
Patients receiving lipid-lowering treatment (*n* = 35)				
*CASR*	0.395 (0.065–4.050)	0.857; 0.987	0.341; 0.221	0.884; 0.890
*RXRA*	0.751 (0.162–1.590)		0.613; 0.278	**3E-5; 7.3E-5**
*LXRA*	1.074 (0.071–1.977)			0.381; 0.827
*ENHO*	0.531 (0.055–2.524)			

Significant correlations are indicated using a bold font

After adjustment for RRT duration, dry body weight, and serum albumin level, there were no significant correlations between relative *CASR* transcript amounts and serum lipids or the TG/HDL-cholesterol ratio. A positive correlation was demonstrated between *ENHO* transcript and LDL-cholesterol in the entire studied group (*P*-value 0.023), and a negative correlation with TG among HD patients not receiving lipid-lowering medication (*P*-value 0.028) (Additional file 1: Table S13). In patients taking lipid-modifying medicines, there were positive correlations between *ENHO* transcript and total cholesterol (*P*-value 0.044) and HDL-cholesterol (*P*-value 0.032).

## Discussion

In the studied HD patients, dyslipidemia occurred in 80.1% of subjects, what is in full agreement with previous reports [1]. In HD subjects like in healthy men [35], a correlation between LDL-cholesterol and non-HDL-cholesterol depended on TG levels, worsening with higher serum TG concentrations.

There were associations between *CASR* SNPs (rs7652589, rs1801725) and dyslipidemia in HD patients not receiving lipid-modifying medications. During treatment with lipid-lowering medications, these correlations are not observed, maybe because not unified protocol of lipid-modifying therapy was used concerning the initiation of such treatment as well as types and doses of medications. Therefore effects of treatment could be hardly comparable concerning *CASR* SNPs.

HD patients bearing the variant allele of *CASR* rs7652589 showed approximately 1.5-fold higher frequency of the atherogenic index equal to or exceeding 3.8, that is values assigned to atherogenic dyslipidemia [2], more elevated TG/HDL-cholesterol ratio, and higher serum TG concentrations. It is worthy to note that the relationship between rs7652589 and atherogenic dyslipidemia was also significant among clinical and laboratory variables tested together. Mentioned above associations were not accompanied by a higher prevalence of CAD in subject harboring the variant allele of rs7652589 SNP. Although atherogenic serum lipid profile is associated with atherosclerosis [36] and CAD [37], our previous retrospective observational study [7] and the 7-year prospective study [38] did not show an influence of *CASR* rs7652589 on all-cause, cardiac or cardiovascular mortality of HD patients, what could be expected for patients bearing the variant allele of rs7652589 that is associated with atherogenic dyslipidemia.

SNP rs1801725 was not directly associated with concentrations of individual lipid components, but the simultaneous occurrence of non-HDL-cholesterol ≥130 mg/dL and TG ≥ 200 mg/dL was showed with about 1.7-fold lower frequency among bearers of the variant allele not receiving lipid-lowering medications. The level of non-HDL-cholesterol is used as a surrogate for increased remnant lipoproteins and apolipoprotein B, at least in normolipidemic individuals [35]. If TG levels are high (≥ 200 mg/dL), much of the non-HDL-cholesterol is very-low-density lipoprotein and intermediate-density lipoprotein remnants, but an association with apolipoprotein B is less strong [39]. In this study, LDL-cholesterol yielded lower correlation with non-HDL-cholesterol at higher serum TG levels. However, further studies might have been recommended to answer whether directly determined circulating lipoprotein remnants, and apolipoprotein B correlate with rs1801725 in HD patients. The variant allele of *CASR* rs1801725, which was associated independently with the more favorable serum lipid profile (the lower coincidence of non-HDL-cholesterol ≥130 mg/dL and TG ≥ 200 mg/dL), showed a borderline negative correlation with *CASR* transcript level additionally. A significance of this finding concerning dyslipidemia and its atherosclerotic consequences is unknown. *CASR* transcript and dry bodyweight show a positive correlation. Subjects with obesity defined by BMI > 30 kg/m^2^ expressed higher levels of *CASR* transcript in the liver, and CaSR was proposed as a contributor to obesity-associated hepatic metabolic consequences [14]. Maybe, lower levels of *CASR* transcript in subjects harboring the variant allele of rs1801725 contribute to the more favorable metabolic profile in HD patients, but direct associations between *CASR* transcript, types of dyslipidemia, CAD, and serum lipids were not found in our study.

The regulatory region of the human *CASR* (chromosome 3q14.3–21) includes two promoters (promoter 1 and 2) that encode for two alternative 5′-untranslated regions. *CASR* has seven exons [40]. Because *CASR* rs1801725 SNP is located in exon 7, it probably cannot directly affect the binding of transcription factors involved in dyslipidemia and CAD as it was shown for rs7652589 SNP [7]. The exonic SNP rs1801725 might be acting through the nonsynonymous exchange of alanine to serine at position 986 in the CaSR cytoplasmic tail. This exchange was initially referred to as associated with the production of a less active receptor by the variant allele of rs1801725 [41, 42]. However, two functional studies documented the normal activity of CaSR coded by the variant allele of rs1801725 SNP [43, 44]. Therefore, the impact of the variant rs1801725 allele cannot be simply explained by a less active CaSR. In our study, the nonsynonymous exchange of alanine to serine was shown together with lower relative *CASR* transcript amount at a borderline level of significance in the dominant model of inheritance in univariate analysis. However, in general, amino acid substitution alters the function (quality) of proteins but not the amount. Thus, lower relative *CASR* transcript amount could not be explained by an impact of rs1801725. Vezzoli et al. [10] designed two constructs containing A (major) or G (variant) allele at the rs6776158 SNP in the *CASR* promoter 1 and found that promoter 1 including the G allele showed lower transcriptional activity than that with the A allele. In light of this finding, it seems reasonable to investigate in further studies the role of rs6776158 and haplotypes formed by rs6776158 and rs1801725 variants concerning dyslipidemia and related comorbidities in HD patients.

It has to be stressed, however, that the fifth-class associations by the BADGE system [30], like those between *CASR* rs7652589 and atherogenic dyslipidemia, *CASR* rs1801725 and dyslipidemia diagnosed by non-HDL-cholesterol ≥130 mg/dL and TG ≥ 200 mg/dL, and *CASR* rs1801725 and CAD prevalence, do not provide assurance of reproducibility. Further studies are needed to confirm direct associations between *CASR* SNPs, serum lipid abnormalities, and CAD.

RXR heterodimerizes with PPARɣ [19], which was found to be activated by up-regulation of CaSR [18]. This co-action may explain the positive correlation between *CASR* and *RXRA* transcripts. RXRα and LXRα form the heterodimer LXRα-RXRα [20]. Thus *RXRA* and *LXRA* transcripts may positively correlate, as we have shown in HD patients. However, much stronger was a positive correlation between *RXRA* and *ENHO* transcripts. In HD subjects, associations were found between *ENHO* rs2281997 and dyslipidemia [22]. In this study, *ENHO* SNPs (rs2281997, rs72735260) did not correlate with *ENHO* transcript levels. It may suggest an association of *ENHO* transcript with other *ENHO* SNP(s) than rs2281997 or rs72735260. However, serum LDL-cholesterol positively correlated with *ENHO* transcript and *ENHO* rs2281997 and *RXRA* rs749759 showed epistatic interaction concerning dyslipidemia diagnosed by LDL-cholesterol ≥100 mg/L. Up-regulation of *ENHO* under dyslipidemic conditions is expected to increase the production of adropin, a protein product of *ENHO*, which is a factor governing glucose and lipid homeostasis [21]. In HD patients, homozygotes of the major allele in *ENHO* rs2281997 were suggested to have higher circulating adropin [27]. In Behçet’s disease, serum adropin level correlated positively with LDL-cholesterol [45]. Therefore, *ENHO* transcript level and adropin were both found as positively associated with LDL-cholesterol. In patients not receiving lipid-lowering treatment, adjustment for body mass, serum albumin level and RRT duration revealed correlation also between *CASR* and *ENHO* transcripts.

Kumar et al. [21] have shown that adropin is associated with suppression of lipogenic gene expression. Inversely, stimulation of LXRα suppresses hepatic *ENHO* expression [21]. We have determined transcripts of tested genes in PBMCs, not in the liver, and demonstrated a borderline negative association between *ENHO* transcript levels and *LXRA* transcripts in unadjusted analyses, what, however, is in agreement with findings of Kumar et al. [21]. *ENHO* transcript levels negatively correlated with serum TG in the studied HD group and subjects with Behçet’s disease [45] as well as plasma adropin negatively associated with TG and atherogenic index in our previous study on HD patients [22]. However, *CASR* transcript showed a weaker association with *ENHO* transcript than that which was found for *RXRA* and *LXRA* transcripts.

Advanced secondary hyperparathyroidism, atherosclerotic plaques, and vascular calcifications frequently occur together in uremic patients [3]. As demonstrated in Additional file 1: Table S2 and Additional file 1: Table S3, higher serum parathyroid hormone (PTH) levels were shown in HD patients with atherogenic dyslipidemia compared with PTH concentrations in HD subjects without this type of dyslipidemia, however, only at the borderline level of significance (*P* = 0.067). In the study by Mitwalli et al. [46], hyperparathyroid dialysis patients had significantly higher serum TG levels compared with subjects without hyperparathyroidism what speaks in favor for the correlation between secondary hyperparathyroidism and atherogenic dyslipidemia.

We previously found that homozygosity in the variant allele (A) of *CASR* polymorphism rs7652589 is associated with more severe secondary hyperparathyroidism [7]. In this study, carriers of the rs7652589 variant allele showed a higher risk of dyslipidemia diagnosed using the atherogenic index. Therefore, secondary hyperparathyroidism and atherogenic dyslipidemia have a common genetic background as *CASR* polymorphism rs7652589. Our study implicates that activating CaSR for treatment of secondary hyperparathyroidism, we may suspect that also the severity of atherogenic dyslipidemia will be ameliorated, at least in subjects harboring the rs7652589 risk allele. Serum lipid profile is worth to be monitored during calcimimetic administration, the best in patients previously genotyped for *CASR* SNPs.

On the other hand, secondary hyperparathyroidism aggravates with prolongation of dialysis treatment [47]. Inversely, serum lipid profile is not worse, if not slightly better, in patients treated with HD > 5 years compared with lipid profile in subjects dialyzed < 1 year [46]. In the group tested for *CASR* transcript, median serum PTH concentration was 2.2-fold higher in patients being on RRT > 5 years compared with PTH in subjects being on RRT < 1 year. This finding occurs together with 2.6-fold higher relative *CASR* transcript level in patients being on RRT > 5 years. However, *CASR* transcript in PBMCs was not associated either with PTH or serum lipids (total cholesterol, HDL-cholesterol, LDL-cholesterol, TG). Higher relative *CASR* transcript levels may not correspond with CaSR activation and function, because mRNA *CASR* maturation and stability, translation, insertion of the CaSR protein into cell membrane, and CaSR turnover may be disturbed as postulated by Garner et al. [48] basing on their study on parathyroid adenomas from patients with primary hyperparathyroidism. In their study, there were no correlations between parathyroid mRNA *CASR* and serum PTH or ionized calcium concentrations [48]. Clinically, aggravation of secondary hyperparathyroidism in long-term dialysis patients is followed by more frequent use of calcimimetics for the amelioration of hyperparathyroidism consequences [49].

Our study did not include HD patients receiving calcimimetic medication. Cinacalcet decreased TG content in adipocytes (line LS14 derived from a human metastatic liposarcoma) by 20% through CaSR activation but CaSR stimulation in HepG2 cells exhibited a 19% increased TG content in the presence of oleic acid and elevation in the expression of proinflammatory factors [14]. In omental adipose tissue obtained from individuals without end-stage renal disease, the *CASR* rs1042636 major homozygosity (AA) was associated with a lower frequency of CaSR responsiveness to the antilipolytic effect of cinacalcet, whereas the rs1042636 variant allele (G) was associated with a greater antilipolytic frequency. Therefore, a suppressive action of cinacalcet on free fatty acid release may be less pronounced in the rs1042636 AA homozygotes. An analysis of the same study samples performed concerning the rs1801725 genotypes yielded no conclusive results [17]. In our study, *CASR* rs7652589 and rs1801725 correlated with dyslipidemia in HD patients but their simultaneous associations with calcimimetics need to be shown in future studies.

## Conclusions

1. In HD patients, *CASR* polymorphisms (rs7652589, rs1801725) play a noticeable role in dyslipidemia. *CASR* is associated with *RXRA*, *LXRA*, and *ENHO* at the transcript level.

2. Relative *CASR* transcript level positively correlates with dry body weight, *RXRA*, *LXRA* and *ENHO* transcripts, and RRT duration, but is not dependent on gender, age, diabetic nephropathy, types of dyslipidemia, or lipid-modifying treatment.

3. Further investigations may elucidate whether other *CASR* SNPs contribute to associations shown in this study.

## Supplementary information

**Additional file 1. **Supplementary material (tables and figures) contains characteristics of the analyzed polymorphisms, data of patients, associations between *CASR* polymorphisms and haplotypes with dyslipidemia, correlates of *CASR*, *RXRA*, *LXRA*, and *ENHO* transcript amounts, the LD data, and correlations between serum cholesterols and serum TG levels.

## Data Availability

The datasets used and/or analyzed during the current study are available from the corresponding author on reasonable request.

## Abbreviations

ALP: Alkaline phosphatase
BADGE: Better Associations for Disease and GEnes
BMI: Body mass index
CAD: Coronary artery disease,
*CASR*: Calcium-sensing receptor gene
CaSR: Calcium-sensing receptor
*ENHO*: Energy homeostasis-associated gene
FDR: False discovery rate
HD: Hemodialysis
HDL: High-density lipoprotein
HRM: High-resolution melting curve
HWE: Hardy-Weinberg equilibrium
K/DOQI: National Kidney Foundation/Kidney Disease Outcomes Quality Initiative
LD: Linkage disequilibrium
LDL: Low-density lipoprotein
LXR: Liver X receptor
*LXRA*: Liver X receptor α gene
MAF: Variant allele frequency
MI: Myocardial infarction
PBMC: Peripheral blood mononuclear cel
PPARɣ: Peroxisome proliferator-activated receptor ɣ
RRT: Renal replacement therapy
RXR: Retinoid X receptor
*RXRA*: Retinoid X receptor α gene
SNP: Single nucleotide polymorphism
TC: Total cholesterol
TG: Triglycerides
