# Supplementary material for: Calcium-sensing receptor gene (CASR) polymorphisms and CASR transcript level concerning dyslipidemia in hemodialysis patients: a cross-sectional study
Source: BMC Nephrol. 2019 Nov 27;20:436. doi: 10.1186/s12882-019-1619-0 (PMC6882244; doi:10.1186/s12882-019-1619-0)
Supplement: Supplementary file 1 — Additional file 1. Supplementary material (tables and figures) contains characteristics of the analyzed polymorphisms, data of patients, associations between CASR polymorphisms and haplotypes with dyslipidemia, correlates of CASR, RXRA, LXRA, and ENHO transcript amounts, the LD data, and correlations between serum cholesterols and serum TG levels. [file 12882_2019_1619_MOESM1_ESM.docx]

**Calcium-sensing receptor gene (*CASR*) polymorphisms and *CASR* transcript level concerning dyslipidemia in hemodialysis patients:
A cross-sectional study**

Alicja E. Grzegorzewska^1^*, Bartosz A. Frycz^2^, Monika Świderska^1^, Leszek Niepolski^3^, Adrianna Mostowska^2^, Paweł P. Jagodziński^2^

^1^ Department of Nephrology, Transplantology and Internal Diseases, Poznan University of Medical Sciences, Poznań, Poland

^2^ Department of Biochemistry and Molecular Biology, Poznan University of Medical Sciences, Poznań, Poland

^3^ B.Braun Avitum Poland, Dialysis Center, Nowy Tomyśl, Poland

^*^Corresponding author:

E-mail: [alicja_grzegorzewska@yahoo.com](mailto:alicja_grzegorzewska@yahoo.com) (AEG)

Supplementary material contains characteristics of the analyzed polymorphisms, data of patients, associations between *CASR* polymorphisms and haplotypes with dyslipidemia, correlates of *CASR*, *RXRA*, *LXRA*, and *ENHO* transcript amounts, the Linkage Disequilibrium data, and correlations between serum cholesterols and serum TG levels.

**Supporting Information**

**Supplementary Tables**

**S1 Table. Characteristics of the analyzed polymorphisms**

| Gene symbol | rs no. | Location^a^ | SNP function^b^ | Alleles^c^ | MAF^d^ | MAF^e^ |
| --- | --- | --- | --- | --- | --- | --- |
| *CASR* | rs7652589 | chr3:122170241 | N/A | A / G | 0.359 | 0.360 |
| *CASR* | rs1801725 | chr3:122284910 | missense (p.Ala986Ser) | G / T | 0.145 | 0.133 |
| *ENHO* | rs2281997 | chr9:34521869 | Intron | C / T | 0.353 | 0.354 |
| *ENHO* | rs72735260 | chr9:34521190 | UTR-3 | G / T | 0.098 | NA |
| *RXRA* | rs10881578 | chr9:134340689 | Intron | A / G | 0.289 | 0.239 |
| *RXRA* | rs10776909 | chr9:134396900 | Intron | C / T | 0.215 | 0.208 |
| *RXRA* | rs749759 | chr9:134432806 | Intron | A / G | 0.234 | 0.208 |
| *LXRA* | rs2279238 | chr11:47260473 | cds-synon | A / G | 0.152 | 0.146 |
| *LXRA* | rs7120118 | chr11:47264739 | Intron | C / T | 0.297 | 0.301 |
| *LXRA* | rs11039155 | chr11:47259211 | UTR-5 | A / G | 0.143 | NA |
|  |  |  |  |  |  |  |

a - Genome assembly GRCh38/hg38

b - According to the Single Nucleotide Polymorphism database (dbSNP)

c - Underline denotes the variant allele.

d - MAF, minor allele frequency (1000 Genomes project, EUR samples)

e - MAF, minor allele frequency (HapMap, CEU samples

**S2 Table. Demographic, clinical and laboratory data of HD patients stratified by serum lipid status**

| Parameter | Patients with dyslipidemia | Patients without dyslipidemia | Patients with dyslipidemia | Patients without dyslipidemia | Non-dyslipidemic patients without antilipemic medication |
| --- | --- | --- | --- | --- | --- |
|  | by K/DOQI criteria by atherogenic index | | | |  |
|  | Group 1 | Group 2 | Group 3 | Group 4 | Group 5 |
| **Demographic data** | N = 621 | N = 587 | N = 576 | N = 632 | N = 240 |
| Male gender, n, % of all | 309 (49.8) | 376 (64.1) | 331 (57.5) | 354 (56.0) | 147 (61.3) |
| Age, years | 66.3 (13.6 - 95.9) | 67.4 (17.1 - 96.1) | 65.6 (17.1 - 91.3) | 68.4 (13.6 - 96.1) | 68.6 (22.3 – 96.1) |
| **Clinical data** |  |  |  |  |  |
| Diabetic nephropathy, n, % of all | 163 (26.2) | 194 (33) | 172 (29.9) | 185 (29.3) | 66 (27.5) |
| Hypertensive/atherosclerotic nephropathy, n, % of all | 128 (20.6) | 111 (18.9) | 113 (19.6) | 126 (19.9) | 49 (20.4) |
| Coronary artery disease, n, % of all^a^ | 219/608 (36.0) | 233/560 (41.6) | 240/556 (43.2) | 212/612 (34.6) | 76/230 (33.0) |
| RRT duration, years | 5.47 (0.01 – 32.01) | 5.59 (0.09 - 30.61) | 5.66 (0.01 - 29.88) | 5.42 (0.01 – 32.01) | 5.44 (0.37 – 30.61) |
| PD prior to HD initiation, n, % of all | 20 (3.2) | 14 (2.4) | 12 (2.1) | 22 (3.5) | 6 (2.5) |
| HD re-started after graft loss, n, % of all | 47 (7.6) | 45 (7.7) | 51 (8.9) | 41 (6.5) | 16 (6.7) |
| Lipid-lowering treatment, n, % of all | 258 (41.6) | 234 (39.9) | 272 (47.2) | 220 (34.8) | N/A |
| Dry body mass, kg | 74 (34.5 – 122.5) | 71.6 (36 – 140) | 77.2 (36 – 140) | 70 (34.5 – 114.5) | 70 (39.2 – 114.5) |
| Body mass index, kg/m^2^ | 26.2 (12.8 – 39.8) | 25.2 (14.3 – 44.2) | 27.1 (14.3 – 44.2) | 24.8 (12.8 – 38.3) | 24.0 (14.7 – 37.4) |
| **Laboratory data** |  |  |  |  |  |
| HDL-cholesterol, mg/dL | 40 (10 - 103) | 40 (5 - 118) | 34 (5 – 71.5) | 47 (13 - 118) | 46 (13 – 118) |
| Triglycerides, mg/dL | 177 (26 - 856) | 123 (29.8 - 1363) | 210 (46 - 1363) | 108 (26 - 260) | 99 (35 – 216) |
| LDL-cholesterol, mg/dL | 120.6 (41.8 - 512) | 71.5 (13.3 - 99.5) | 101 (17 - 512) | 91 (13.3 - 255) | 73.3 (20 – 99.5) |
| Non-HDL-cholesterol, mg/dL | 159 (104 - 593) | 98 (27 - 202) | 146 (44 - 593) | 114 (27 - 282) | 94 (27 – 202) |
| TG/HDL-cholesterol ratio | 4.42 (0.43 – 25.88) | 3.13 (0.44 – 49.71) | 5.99 (3.81 – 49.71) | 2.49 (0.43 – 3.79) | 2.28 (0.44 – 3.77) |
| ALT, IU/L | 14 (0.6 - 135) | 14 (2 - 195) | 14 (2 - 195) | 14 (0.6 – 135) | 13 (2 – 107) |
| ALP, IU/L | 93.8 (13.5 – 1684) | 100 (15 – 1408) | 95.5 (13.5 – 1408) | 98 (15 – 1684) | 109 (15 – 1353.3) |
| Total calcium, mg/dL | 8.84 (6.01 – 12.80) | 8.84 (4.51 – 12.77) | 8.82 (6.01 – 12.80) | 8.85 (4.51 – 12.80) | 8.80 (5.40 – 12.77) |
| Phosphorus, mg/dL | 5.06 (2.32 – 11.99) | 5.07 (1.75 – 10.96) | 5.18 (1.95 – 11.27) | 5.00 (1.75 – 11.99) | 5.01 (2.40 – 9.45) |
| PTH, pg/mL | 392 (7.3 - 3757) | 359.8 (13.7 - 2991.5) | 409.1 (8.9 - 3206) | 353.2 (7.3 – 3757) | 355.1 (21.5 – 2991.5) |

a – data obtained in 1168 patients

Abbreviations: ALT - alanine aminotransferase, ALP – alkaline phosphatase, LF-HD – low-flux hemodialysis, HD – hemodialysis, HDL – high-density lipoprotein, LDL – low-density lipoprotein, N/A – not applicable, PD – peritoneal dialysis, PTH - parathyroid hormone, RRT - renal replacement therapy, TG - triglycerides

Conversion factors to SI units are as follows: for alanine aminotransferase – 1 U/L = 0.0167 µkat/L, for alkaline phosphatase – 1 U/L = 0.0167 µkat/L, for calcium – 1 mg/dL = 0.2495 mmol/L, for cholesterols – 1 mg/dL = 0.0259 mmol/L, for triglycerides – 1 mg/dL = 0.0113 mmol/L, parathyroid hormone – 1 pg/mL = 0.1061 pmol/L, and phosphorus – 1 mg/dL = 0.3229 mmol/L

**S3 Table.** Statistical analysis of data shown in S2 Table among groups 1 – 5 of HD patients stratified by serum lipemic status

| Parameter | P-value for compared groups^a^ | | | |
| --- | --- | --- | --- | --- |
|  | 1 vs. 2 | 1 vs. 5 | 3 vs. 4 | 3 vs. 5 |
| **Demographic data** |  |  |  |  |
| Male gender, n, % of all | **1.9E-8** | **0.002** | 0.611 | 0.317 |
| Age, years | 0.893 | 0.852 | **0.009** | 0.284 |
| **Clinical data** |  |  |  |  |
| Diabetic nephropathy, n, % of all | **0.010** | 0.709 | 0.823 | 0.499 |
| Coronary artery disease, n, % of all | 0.050 | 0.421 | **0.003** | **0.008** |
| Hypertensive/atherosclerotic nephropathy, n, % of all | 0.458 | 0.949 | 0.890 | 0.794 |
| RRT duration, years | 0.987 | 0.531 | 0.300 | 0.308 |
| HD re-started after graft loss, n, % of all | 0.949 | 0.649 | 0.121 | 0.300 |
| Antilipemic treatment, n, % of all | 0.552 | **<2.2E-16** | **1.2E-5** | **<2.2E-16** |
| Dry body mass, kg | 0.099 | **0.0008** | **1.4E-8** | **1.0E-6** |
| Body mass index, kg/m^2^ | **0.002** | **3.2E-6** | **3.9E-10** | **5.3E-9** |
| **Laboratory data** |  |  |  |  |
| ALT, IU/L | 0.604 | 0.783 | 0.248 | 0.358 |
| ALP, IU/L | **0.010** | **0.0001** | 0.304 | **0.0008** |
| Total calcium, mg/dL | 0.969 | 0.249 | 0.850 | 0.220 |
| Phosphorus, mg/dL | 0.979 | 0.737 | 0.176 | 0.373 |
| PTH, pg/mL | 0.213 | 0.488 | 0.067 | 0.343 |

Significant differences are indicated using bold font.

a - Mann-Whitney U test was used for comparison of continuous variables. Chi-square test was applied for comparison of dichotomous variables

**S4 Table. Statistical analyses of associations between *CASR* rs7652589 and dyslipidemia**

| Patient group | Dyslipidemia, n | Model of inheritance | Comparison with a group without dyslipidemia by a respective criterion | | Comparison with a non-dyslipidemic group without antilipemic medication, n = 219 | |
| --- | --- | --- | --- | --- | --- | --- |
|  |  |  | OR (95% CI) | P-value^a^ | OR (95% CI) | P-value^a^ |
| All | by K/DOQI criteria, atherogenic index, or both among subjects not receiving lipid-lowering medication together with all patients treated with antilipemic medications  n = 920 of 1139 | dominant | N/A | N/A | 1.277 (0.946 - 1.723) | 0.109 |
|  |  | recessive | N/A | N/A | 0.819 (0.545 - 1.232) | 0.337 |
|  |  | additive | N/A | N/A | 0.978 (0.630 - 1.518) | 0.920 |
|  | by K/DOQI criteria,  n = 597 of 1139 | dominant | 0.962 (0.756 - 1.222) | 0.749 | 1.195 (0.872 - 1.636) | 0.268 |
|  |  | recessive | 0.783 (0.560 - 1.096) | 0.153 | 0.755 (0.489 - 1.167) | 0.205 |
|  |  | additive | 0.791 (0.549 - 1.139) | 0.207 | 0.874 (0.547 - 1.396) | 0.573 |
|  | by plasma LDL-cholesterol concentration  ≥ 100 mg/dL  n = 532 of 1139 | dominant | 0.983 (0.773 - 1.249) | 0.887 | 1.206 (0.876 - 1.661) | 0.252 |
|  |  | recessive | 0.908 (0.649 - 1.272) | 0.576 | 0.810 (0.522 - 1.256) | 0.346 |
|  |  | additive | 0.911 (0.632 - 1.313) | 0.616 | 0.934 (0.582 - 1.500) | 0.777 |
|  | by non-HDL-cholesterol  ≥ 130 mg/dL and TG ≥ 200 mg/dL  n = 236 of 1139 | dominant | 0.936 (0.698 - 1.257) | 0.661 | 1.155 (0.794 - 1.680) | 0.450 |
|  |  | recessive | 0.755 (0.485 - 1.173) | 0.210 | 0.679 (0.396 - 1.165) | 0.158 |
|  |  | additive | 0.751 (0.468 - 1.207) | 0.236 | 0.780 (0.437 - 1.391) | 0.399 |
|  | by atherogenic index,  n = 540 of 1139 | dominant | 1.177 (0.926 - 1.497) | 0.184 | 1.327 (0.963 - 1.829) | 0.083 |
|  |  | recessive | 0.989 (0.707 - 1.383) | 0.948 | 0.848 (0.548 - 1.311) | 0.458 |
|  |  | additive | 1.096 (0.761 - 1.579) | 0.621 | 1.033 (0.645 - 1.654) | 0.894 |
| Patients in whom dyslipidemia was abolished by antilipemic medication  excluded | by K/DOQI criteria,  n = 597 of 918 | dominant | 1.059 (0.802 - 1.399) | 0.684 | 1.195 (0.872 - 1.636) | 0.268 |
|  |  | recessive | 0.727 (0.496 - 1.064) | 0.100 | 0.755 (0.489 - 1.167) | 0.205 |
|  |  | additive | 0.788 (0.521 - 1.192) | 0.259 | 0.874 (0.547 - 1.396) | 0.573 |
|  | by plasma LDL-cholesterol concentration ≥ 100 mg/dL  n = 532 of 890 | dominant | 1.037 (0.787 - 1.366) | 0.797 | 1.206 (0.876 - 1.661) | 0.252 |
|  |  | recessive | 0.848 (0.580 - 1.242) | 0.397 | 0.810 (0.522 - 1.256) | 0.346 |
|  |  | additive | 0.888 (0.588 - 1.343) | 0.574 | 0.934 (0.582 - 1.500) | 0.777 |
|  | by non-HDL-cholesterol  ≥ 130 mg/dL and TG ≥ 200 mg/dL  n = 236 of 791 | Dominant | 0.990 (0.724 - 1.353) | 0.949 | 1.155 (0.794 - 1.680) | 0.450 |
|  |  | Recessive | 0.705 (0.444 - 1.118) | 0.136 | 0.679 (0.396 - 1.165) | 0.158 |
|  |  | additive | 0.734 (0.447 - 1.205) | 0.221 | 0.780 (0.437 - 1.391) | 0.399 |
|  | by atherogenic index,  n = 540 of 927 | dominant | 1.254 (0.959 - 1.640) | 0.098 | 1.327 (0.963 - 1.829) | 0.083 |
|  |  | recessive | 0.974 (0.669 - 1.417) | 0.889 | 0.848 (0.548 - 1.311) | 0.458 |
|  |  | additive | 1.123 (0.748 - 1.687) | 0.574 | 1.033 (0.645 - 1.654) | 0.894 |
| Only patients not receiving antilipemic medication | by K/DOQI criteria,  n = 345 of 666 | dominant | 1.182 (0.863 - 1.617) | 0.298 | 1.332 (0.942 - 1.885) | 0.105 |
|  |  | recessive | 0.720 (0.466 - 1.112) | 0.137 | 0.749 (0.462 - 1.213) | 0.238 |
|  |  | additive | 0.838 (0.522 - 1.344) | 0.462 | 0.929 (0.552 - 1.564) | 0.782 |
|  | by plasma LDL-cholesterol concentration ≥ 100 mg/dL  n = 308 of 666 | dominant | 1.126 (0.822 - 1.544) | 0.459 | 1.310 (0.919 - 1.868) | 0.136 |
|  |  | recessive | 0.846 (0.547 - 1.309) | 0.452 | 0.807 (0.495 - 1.316) | 0.390 |
|  |  | additive | 0.933 (0.580 - 1.501) | 0.777 | 0.981 (0.579 - 1.665) | 0.945 |
|  | by non-HDL-cholesterol  ≥ 130 mg/dL and TG ≥ 200 mg/dL  n = 111 of 666 | dominant | 1.373 (0.889 - 2.122) | 0.152 | 1.603 (0.990 - 2.595) | 0.054 |
|  |  | recessive | 0.540 (0.271 - 1.076) | 0.076 | 0.521 (0.248 - 1.095) | 0.081 |
|  |  | additive | 0.714 (0.339 - 1.506) | 0.375 | 0.759 (0.340 - 1.695) | 0.501 |
|  | by atherogenic index,  n = 279 of 666 | dominant | 1.441 (1.044 - 1.988) | 0.026 | 1.525 (1.057 - 2.200) | 0.024 |
|  |  | recessive | 1.040 (0.672 - 1.610) | 0.861 | 0.906 (0.555 - 1.479) | 0.692 |
|  |  | additive | 1.302 (0.806 - 2.104) | 0.279 | 1.197 (0.701 - 2.046) | 0.510 |
| Only patients receiving antilipemic medication | by K/DOQI criteria,  n = 252 of 473 | dominant | 0.719 (0.495 - 1.046) | 0.084 | 1.033 (0.716 - 1.491) | 0.861 |
|  |  | recessive | 0.891 (0.524 -1.516) | 0.671 | 0.765 (0.456 -1.283) | 0.309 |
|  |  | additive | 0.737 (0.415 - 1.312) | 0.299 | 0.810 (0.465 - 1.410) | 0.456 |
|  | by plasma LDL-cholesterol concentration ≥ 100 mg/dL  n 224 = of 473 | dominant | 0.813 (0.561 - 1.179) | 0.275 | 1.079 (0.739 - 1.574) | 0.694 |
|  |  | recessive | 1.012 (0.595 -1.721) | 0.964 | 0.813 (0.480 -1.378) | 0.441 |
|  |  | additive | 0.889 (0.501 - 1.579) | 0.688 | 0.876 (0.497 - 1.544) | 0.647 |
|  | by non-HDL-cholesterol  ≥ 130 mg/dL and TG ≥ 200 mg/dL  n = 125 of 473 | dominant | 0.652 (0.431 - 0.988) | 0.043 | 0.881 (0.566 - 1.370) | 0.573 |
|  |  | recessive | 1.033 (0.568 - 1.879) | 0.914 | 0.828 (0.442 - 1.548) | 0.553 |
|  |  | additive | 0.797 (0.421 - 1.511) | 0.487 | 0.792 (0.407 - 1.544) | 0.494 |
|  | by atherogenic index,  n = 261 of 473 | dominant | 0.907 (0.624 - 1.319) | 0.610 | 1.151 (0.798 - 1.658) | 0.452 |
|  |  | recessive | 0.945 (0.555 -1.609) | 0.836 | 0.787 (0.473 -1.312) | 0.358 |
|  |  | additive | 0.897 (0.504 - 1.596) | 0.710 | 0.886 (0.511 - 1.534) | 0.665 |

a – Pearson's chi-squared test

Abbreviations: K/DOQI – Kidney Disease Outcomes Quality Initiative, LDL-cholesterol – low density lipoprotein cholesterol, N/A – not applicable

**S5 Table. Statistical analyses of associations between *CASR* rs1801725 and dyslipidemia**

| Patient group | Dyslipidemia, n | Model of inheritance | Comparison with a group without dyslipidemia by a respective criterion | | Comparison with a non-dyslipidemic group without antilipemic medication, n = 232 | |
| --- | --- | --- | --- | --- | --- | --- |
|  |  |  | OR (95% CI) | P-value^a^ | OR (95% CI) | P-value^a^ |
| All | by K/DOQI criteria, atherogenic index, or both among subjects not receiving lipid-lowering medication together with all patients treated with antilipemic medications  n = 927 of 1159 | dominant | N/A | N/A | 0.938 (0.687 - 1.279) | 0.685 |
|  |  | recessive | N/A | N/A | 0.746 (0.293 - 1.901) | 0.605^b^ |
|  |  | additive | N/A | N/A | 0.736 (0.288 - 1.885) | 0.602^b^ |
|  | by K/DOQI criteria,  n = 599 of 1159 | dominant | 0.784 (0.610 - 1.007) | 0.056 | 0.842 (0.606 - 1.171) | 0.306 |
|  |  | recessive | 0.787 (0.350 - 1.772) | 0.562 | 0.705 (0.258 - 1.928) | 0.585^b^ |
|  |  | additive | 0.735 (0.325 - 1.659) | 0.456 | 0.675 (0.246 - 1.855) | 0.419^b^ |
|  | by plasma LDL-cholesterol concentration ≥ 100 mg/dL  n = 529 of 1159 | dominant | 0.896 (0.696 - 1.153) | 0.393 | 0.894 (0.640 - 1.250) | 0.514 |
|  |  | recessive | 1.008 (0.448 - 2.269) | 0.985 | 0.800 (0.292 - 2.189) | 0.663 |
|  |  | additive | 0.975 (0.432 - 2.202) | 0.951 | 0.777 (0.283 - 2.138) | 0.625 |
|  | by non-HDL-cholesterol ≥ 130 mg/dL and TG ≥ 200 mg/dL  n = 243 of 1159 | dominant | 0.720 (0.522 - 0.993) | 0.045 | 0.730 (0.489 - 1.090) | 0.124 |
|  |  | recessive | 0.533 (0.158 - 1.801) | 0.303 | 0.471 (0.116 - 1.905) | 0.329^b^ |
|  |  | additive | 0.491 (0.145 - 1.663) | 0.243 | 0.437 (0.108 - 1.775) | 0.316^b^ |
|  | by atherogenic index,  n = 548 of 1159 | dominant | 1.060 (0.825 - 1.362) | 0.648 | 0.980 (0.704 - 1.364) | 0.903 |
|  |  | recessive | 0.551 (0.234 - 1.298) | 0.167 | 0.558 (0.191 - 1.627) | 0.375^b^ |
|  |  | additive | 0.567 (0.240 - 1.341) | 0.191 | 0.561 (0.192 - 1.643) | 0.374^b^ |
| Patients in whom dyslipidemia was abolished by antilipemic medication  excluded | by K/DOQI criteria,  n = 599 of 939 | dominant | 0.798 (0.598 - 1.064) | 0.124 | 0.842 (0.606 - 1.171) | 0.306 |
|  |  | recessive | 0.776 (0.309 - 1.949) | 0.589 | 0.705 (0.258 - 1.928) | 0.585^b^ |
|  |  | additive | 0.729 (0.289 - 1.838) | 0.501 | 0.675 (0.246 - 1.855) | 0.419^b^ |
|  | by plasma LDL-cholesterol concentration ≥ 100 mg/dL  n = 529 of 911 | dominant | 0.908 (0.682 - 1.209) | 0.507 | 0.894 (0.640 - 1.250) | 0.514 |
|  |  | recessive | 0.993 (0.396 - 2.492) | 0.988 | 0.800 (0.292 - 2.189) | 0.663 |
|  |  | additive | 0.964 (0.383 – 2.430) | 0.939 | 0.777 (0.283 - 2.138) | 0.625 |
|  | by non-HDL-cholesterol ≥ 130 mg/dL and TG ≥ 200 mg/dL  n = 243 of 812 | dominant | 0.707 (0.504 - 0.992) | 0.045 | 0.730 (0.489 - 1.090) | 0.124 |
|  |  | recessive | 0.495 (0.141 - 1.740) | 0.264 | 0.471 (0.116 - 1.905) | 0.329^b^ |
|  |  | additive | 0.454 (0.129 - 1.601) | 0.209 | 0.437 (0.108 - 1.775) | 0.316^b^ |
|  | by atherogenic index,  n = 548 of 949 | dominant | 1.005 (0.760 - 1.327) | 0.974 | 0.980 (0.704 - 1.364) | 0.903 |
|  |  | recessive | 0.579 (0.227 - 1.481) | 0.249 | 0.558 (0.191 - 1.627) | 0.375^b^ |
|  |  | additive | 0.586 (0.228 - 1.505) | 0.262 | 0.561 (0.192 - 1.643) | 0.374^b^ |
| Only patients not receiving antilipemic medication | by K/DOQI criteria,  n = 346 of 686 | dominant | 0.804 (0.580 - 1.114) | 0.189 | 0.848 (0.590 - 1.220) | 0.375 |
|  |  | recessive | 0.732 (0.252 - 2.133) | 0.567 | 0.665 (0.212 - 2.087) | 0.557^b^ |
|  |  | additive | 0.690 (0.236 - 2.018) | 0.496 | 0.639 (0.202 - 2.015) | 0.551^b^ |
|  | by plasma LDL-cholesterol concentration ≥ 100 mg/dL  n = 304 of 686 | dominant | 0.915 (0.659 - 1.271) | 0.596 | 0.902 (0.622 - 1.307) | 0.585 |
|  |  | recessive | 0.941 (0.323 - 2.742) | 0.912 | 0.758 (0.241 - 2.383) | 0.635 |
|  |  | additive | 0.917 (0.314 - 2.685) | 0.875 | 0.740 (0.234 - 2.336) | 0.606 |
|  | by non-HDL-cholesterol ≥ 130 mg/dL and TG ≥ 200 mg/dL  n = 117 of 686 | dominant | 0.573 (0.356 - 0.922) | 0.021 | 0.592 (0.351 - 0.997) | 0.048 |
|  |  | recessive | 0.163 (0.010 - 2.752) | 0.144^b^ | 0.148 (0.008 - 2.655) | 0.185^b^ |
|  |  | additive | 0.144 (0.009 - 2.437) | 0.083^b^ | 0.133 (0.007 - 2.381) | 0.091^b^ |
|  | by atherogenic index,  n = 285 of 686 | dominant | 0.934 (0.670 - 1.300) | 0.684 | 0.910 (0.625 - 1.326) | 0.624 |
|  |  | recessive | 0.557 (0.173 - 1.793) | 0.320 | 0.536 (0.150 - 1.923) | 0.356^b^ |
|  |  | additive | 0.551 (0.171 - 1.783) | 0.313 | 0.527 (0.146 - 1.901) | 0.351^b^ |
| Only patients receiving antilipemic medication | by K/DOQI criteria,  n = 253 of 473 | dominant | 0.755 (0.510 - 1.118) | 0.159 | 0.833 (0.564 - 1.231) | 0.360 |
|  |  | recessive | 0.867 (0.248 - 3.035) | 1.000^b^ | 0.759 (0.229 - 2.522) | 0.652 |
|  |  | additive | 0.798 (0.227 - 2.808) | 0.756^b^ | 0.724 (0.217 - 2.418) | 0.598 |
|  | by plasma LDL-cholesterol concentration ≥ 100 mg/dL  n 225 = of 473 | dominant | 0.869 (0.587 - 1.287) | 0.484 | 0.885 (0.593 - 1.320) | 0.549 |
|  |  | recessive | 1.105 (0.316 - 3.867) | 1.000^b^ | 0.856 (0.258 - 2.846) | 0.800 |
|  |  | additive | 1.056 (0.300 - 3.717) | 1.000^b^ | 0.828 (0.248 - 2.769) | 0.759 |
|  | by non-HDL-cholesterol ≥ 130 mg/dL and TG ≥ 200 mg/dL  n = 126 of 473 | dominant | 0.885 (0.565 - 1.386) | 0.594 | 0.871 (0.542 - 1.402) | 0.570 |
|  |  | recessive | 1.185 (0.302 - 4.653) | 0.730^b^ | 0.919 (0.226 - 3.737) | 1.000^b^ |
|  |  | additive | 1.138 (0.288 - 4.497) | 1.000^b^ | 0.883 (0.216 - 3.617) | 1.000^b^ |
|  | by atherogenic index,  n = 263 of 473 | dominant | 1.273 (0.856 - 1.895) | 0.233 | 1.058 (0.725 - 1.545) | 0.769 |
|  |  | recessive | 0.525 (0.146 - 1.886) | 0.351^b^ | 0.582 (0.162 - 2.087) | 0.527^b^ |
|  |  | additive | 0.573 (0.159 - 2.066) | 0.524^b^ | 0.599 (0.166 - 2.161) | 0.528^b^ |

a – Pearson's chi-squared test; b – Fisher’s test

Abbreviations: K/DOQI – Kidney Disease Outcomes Quality Initiative, LDL-cholesterol – low-density lipoprotein cholesterol, N/A – not applicable

**S6 Table**. *CASR* rs7652589 and prevalence of CAD in HD patients not receiving lipid-lowering medication

| **Genotypes, MAF** | | | **CAD**  **(n, frequency)**  **n = 214** | | **Without CAD**  **(n, frequency)**  **n = 424** | **Odds ratio (95% CI),**  **P-value^a^** | |
| --- | --- | --- | --- | --- | --- | --- | --- |
| *CASR* rs7652589 (P*_trend_*^b^ = 0.654, _P_*_genotype_* ^a^ = 0.904) | | | | | | | |
| GG | 79 (36.9) | | 163 (38.4) | | | **Reference** | |
| GT | 103 (48.1) | | 202 (47.6) | | | 1.052 (0.735 - 1.506), 0.781 | |
| TT | 32 (15.0) | | 59 (13.9) | | | 1.119 (0.674 - 1.859), 0.664 | |
| TT + GT vs GG | 135 (63.1) | | 261 (61.6) | | | 1.067 (0.760 - 1.499), 0.707 | |
| TT vs GG + GT | 32 (15.0) | | 59 (13.9) | | | 1.088 (0.683 - 1.733), 0.723 | |
| MAF | (0.39) | | (0.38) | | | 1.056 (0.832 - 1.340), 0.656 | |
| P-value for HWE | 0.868 | | 0.776 | | |  | |

a – Pearson's Chi-squared test; b – Cochran-Armitage Trend Test

**S7 Table**. *CASR* rs1801725 and prevalence of coronary artery disease (CAD) in HD patients not receiving lipid-lowering medication

| **Genotypes, MAF** | | | **CAD**  **(n, frequency)**  **n = 214** | | **Without CAD**  **(n, frequency)**  **n = 443** | **Odds ratio (95% CI),**  **P-value^a^** | |
| --- | --- | --- | --- | --- | --- | --- | --- |
| *CASR* rs1801725 (P*_trend_*^b^ = 0.057, _P_*_genotype_* ^a ,c^= 0.064) | | | | | | | |
| GG | 157 (73.4) | | 300 (67.7) | | | **Reference** | |
| GT | 56 (26.2) | | 130 (29.3) | | | 0.823 (0.570 - 1.189), 0.300 | |
| TT | 1 (0.5) | | 13 (2.9) | | | 0.147 (0.019 - 1.134), **0.042**^c^ | |
| TT + GT vs GG | 57 (26.6) | | 143 (32.3) | | | 0.762 (0.530 - 1.095), 0.141 | |
| TT vs GG + GT | 1 (0.5) | | 13 (2.9) | | | 0.155 (0.020 - 1.195), **0.044**^c^ | |
| MAF | (0.14) | | (0.18) | | | 0.734 (0.529 - 1.017), 0.062 | |
| P-value for HWE | 0.087 | | 0.810 | | |  | |

a – Pearson's Chi-squared test; b – Cochran-Armitage Trend Test, c – Fisher’s test

**S8 Table**. Associations of *CASR* haplotypes with dyslipidemia in HD patients not treated with lipid-lowering medicines

1. Patients dyslipidemic by K/DOQI vs. patients without dyslipidemia by K/DOQI

| **Gene** | **Polymorphisms** | **Haplotype** | **Freq.** | **Case,Control Frequencies** | **Chi Square** | **P Value** | **P_corr_ Value^a^** |
| --- | --- | --- | --- | --- | --- | --- | --- |
| ***CASR*** | **rs7652589_rs1801725** | GG | 0.516 | 0.509, 0.524 | 0.22 | 0.6389 | 0.9420 |
|  |  | AG | 0.323 | 0.336, 0.306 | 1.054 | 0.3047 | 0.5870 |
|  |  | GT | 0.093 | 0.082, 0.107 | 1.904 | 0.1676 | 0.3270 |
|  |  | AT | 0.069 | 0.073, 0.063 | 0.374 | 0.5406 | 0.8820 |
|  |  |  |  |  |  |  |  |
| ^a^ p value calculated using permutation test and a total of 1,000 permutations. | | | | |  |  |  |

B. Patients dyslipidemic by K/DOQI vs patients without dyslipidemia by all used criteria

| **Gene** | **Polymorphisms** | **Haplotype** | **Freq.** | **Case,Control Frequencies** | **Chi Square** | **P Value** | **P_corr_ Value^a^** |
| --- | --- | --- | --- | --- | --- | --- | --- |
| ***CASR*** | **rs7652589_rs1801725** | GG | 0.521 | 0.521, 0.519 | 0.005 | 0.9430 | 1.0000 |
|  |  | AG | 0.322 | 0.329, 0.311 | 0.461 | 0.4971 | 0.8410 |
|  |  | GT | 0.102 | 0.095, 0.113 | 0.992 | 0.3191 | 0.5910 |
|  |  | AT | 0.056 | 0.054, 0.057 | 0.051 | 0.8212 | 0.9960 |
|  |  |  |  |  |  |  |  |

^a^ p value calculated using permutation test and a total of 1,000 permutations.

C. Patients dyslipidemic by the atherogenic index vs. patients without dyslipidemia by this index

| **Gene** | **Polymorphisms** | **Haplotype** | **Freq.** | **Case,Control Frequencies** | **Chi Square** | **P Value** | **P_corr_ Value^a^** |
| --- | --- | --- | --- | --- | --- | --- | --- |
| ***CASR*** | **rs7652589_rs1801725** | GG | 0.520 | 0.509, 0.528 | 0.517 | 0.4720 | 0.8140 |
|  |  | AG | 0.318 | 0.336, 0.305 | 1.565 | 0.2109 | 0.4250 |
|  |  | GT | 0.096 | 0.083, 0.105 | 1.958 | 0.1618 | 0.3370 |
|  |  | AT | 0.066 | 0.072, 0.062 | 0.576 | 0.4479 | 0.7890 |
|  |  |  |  |  |  |  |  |
| ^a^ p value calculated using permutation test and a total of 1,000 permutations. | | | | |  |  |  |

D. Patients dyslipidemic by the atherogenic index vs. patients without dyslipidemia by all used criteria

| **Gene** | **Polymorphisms** | **Haplotype** | **Freq.** | **Case,Control Frequencies** | **Chi Square** | **P Value** | **P_corr_ Value^a^** |
| --- | --- | --- | --- | --- | --- | --- | --- |
| ***CASR*** | **rs7652589_rs1801725** | GG | 0.516 | 0.509, 0.524 | 0.220 | 0.6389 | 0.9420 |
|  |  | AG | 0.323 | 0.336, 0.306 | 1.054 | 0.3047 | 0.5870 |
|  |  | GT | 0.093 | 0.082, 0.107 | 1.904 | 0.1676 | 0.3270 |
|  |  | AT | 0.069 | 0.073, 0.063 | 0.374 | 0.5406 | 0.8820 |
|  |  |  |  |  |  |  |  |
| ^a^ p value calculated using permutation test and a total of 1,000 permutations | | | | |  |  |  |

E. Patients with dyslipidemia diagnosed by TG ≥200 mg/dL and non-HDL-cholesterol ≥130 mg/dL vs. patients without dyslipidemia by this criterion

| **Gene** | **Polymorphisms** | **Haplotype** | **Freq.** | **Case,Control Frequencies** | **Chi Square** | **P Value** | **P_corr_ Value^a^** |
| --- | --- | --- | --- | --- | --- | --- | --- |
| ***CASR*** | rs7652589_rs1801725 | GG | 0.520 | 0.548, 0.514 | 0.927 | 0.3356 | 0.6300 |
|  |  | AG | 0.318 | 0.344, 0.313 | 0.883 | 0.3475 | 0.6460 |
|  |  | GT | 0.096 | 0.065, 0.102 | 3.376 | 0.0662 | 0.1100 |
|  |  | AT | 0.066 | 0.044, 0.071 | 2.317 | 0.1280 | 0.2340 |
|  |  |  |  |  |  |  |  |
|  |  | ^a^ p value calculated using permutation test and a total of 1,000 permutations | | | | | |

F. Patients with dyslipidemia diagnosed by LDL-cholesterol ≥100 mg/dL vs. patients without dyslipidemia by this criterion

| **Gene** | **Polymorphisms** | **Haplotype** | **Freq.** | **Case,Control Frequencies** | **Chi Square** | **P Value** | **P_corr_ Value^a^** |
| --- | --- | --- | --- | --- | --- | --- | --- |
| ***CASR*** | rs7652589_rs1801725 | GG | 0.520 | 0.523, 0.518 | 0.038 | 0.8457 | 0.9950 |
|  |  | AG | 0.318 | 0.321, 0.316 | 0.03 | 0.8615 | 0.9980 |
|  |  | GT | 0.096 | 0.093, 0.098 | 0.078 | 0.7806 | 0.9850 |
|  |  | AT | 0.066 | 0.063, 0.068 | 0.151 | 0.6975 | 0.9680 |
|  |  |  |  |  |  |  |  |
|  |  | ^a^p value calculated using permutation test and a total of 1,000 permutations | | | | | |

G. Patients with dyslipidemia diagnosed by TG ≥200 mg/dL and non-HDL-cholesterol ≥130 mg/dL vs. patients without dyslipidemia by all used criteria

| **Gene** | **Polymorphisms** | **Haplotype** | **Freq.** | **Case,Control Frequencies** | **Chi Square** | **P Value** | **P_corr_ Value** |
| --- | --- | --- | --- | --- | --- | --- | --- |
| ***CASR*** | rs7652589_rs1801725 | GG | 0.524 | 0.542, 0.515 | 0.487 | 0.4853 | 0.844 |
|  |  | AG | 0.328 | 0.350, 0.316 | 0.901 | 0.3424 | 0.667 |
|  |  | GT | 0.099 | 0.071, 0.114 | 3.494 | 0.0616 | 0.116 |
|  |  | AT | 0.049 | 0.037, 0.055 | 1.178 | 0.2778 | 0.561 |
|  |  |  |  |  |  |  |  |
|  | ^a^p value calculated using permutation test and a total of 1,000 permutations | | | | |  |  |

H. Patients with dyslipidemia diagnosed by LDL-cholesterol ≥100 mg/dL vs. patients without dyslipidemia by all used criteria

| **Gene** | **Polymorphisms** | **Haplotype** | **Freq.** | **Case,Control Frequencies** | **Chi Square** | **P Value** | **P_corr_ Value** |
| --- | --- | --- | --- | --- | --- | --- | --- |
| ***CASR*** | rs7652589_rs1801725 | GG | 0.518 | 0.519, 0.517 | 0.003 | 0.9547 | 1.000 |
|  |  | AG | 0.320 | 0.325, 0.313 | 0.179 | 0.6720 | 0.953 |
|  |  | GT | 0.104 | 0.098, 0.112 | 0.581 | 0.4458 | 0.780 |
|  |  | AT | 0.059 | 0.059, 0.059 | 0.001 | 0.9783 | 1.000 |
|  |  |  |  |  |  |  |  |
|  | ^a^p value calculated using permutation test and a total of 1,000 permutations | | | | |  |  |

**S9 Table.** *CASR* rs1801725 polymorphic variants and serum lipids in HD patients not receiving antilipemic medication (n = 686)

| Parameter | GG | GT | TT | Model  of inheritance | P-value^a^ | Adjusted P-value^b^ |
| --- | --- | --- | --- | --- | --- | --- |
|  | n = 478 | n = 194 | n = 14 |  |  |  |
| Total cholesterol, mg/dL | 172 (65 – 316) | 166 (75 – 363) | 176.3 (92 – 221) | GT + TT vs GG | 0.193 | 0.917 |
|  |  |  |  | TT vs GG + GT | 0.724 | 0.220 |
|  |  |  |  | TT vs GG | 0.644 | 0.197 |
| HDL-cholesterol, mg/dL | 40.9 (10 – 118) | 41 (6 – 103) | 40.5 (17.3 – 68) | GT + TT vs GG | 0.888 | 0.853 |
|  |  |  |  | TT vs GG + GT | 0.887 | 0.236 |
|  |  |  |  | TT vs GG | 0.919 | 0.255 |
| Triglycerides, mg/dL | 137 (35 – 1105) | 138.5 (54 – 691) | 135 (63 – 302) | GT + TT vs GG | 0.930 | 0.322 |
|  |  |  |  | TT vs GG + GT | 0.678 | 0.738 |
|  |  |  |  | TT vs GG | 0.686 | 0.659 |
| LDL-cholesterol, mg/dL | 95.9 (26.8 – 369) | 89.7 (20 – 255) | 90 (44.3 – 135) | GT + TT vs GG | 0.297 | 0.759 |
|  |  |  |  | TT vs GG + GT | 0.536 | 0.445 |
|  |  |  |  | TT vs GG | 0.481 | 0.387 |
| Non-HDL-cholesterol, mg/dL | 128.5 (51 – 279) | 122 (8 – 282) | 129.5 (74.7 – 181) | GT + TT vs GG | 0.409 | 0.899 |
|  |  |  |  | TT vs GG + GT | 0.639 | 0.421 |
|  |  |  |  | TT vs GG | 0.607 | 0.394 |
| TG/HDL-cholesterol ratio | 3.4 (0.4 – 34.5) | 3.5 (0.9 – 30.8) | 3.2 (1.2 – 8.7) | GT + TT vs GG | 0.897 | 0.214 |
|  |  |  |  | TT vs GG + GT | 0.785 | 0.335 |
|  |  |  |  | TT vs GG | 0.815 | 0.297 |

a - Mann Whitney test

b - adjustment for gender, age, BMI, diabetic nephropathy, coronary artery disease, and alkaline phosphatase activity

Conversion factors to SI units are as follows: for cholesterols – 1 mg/dL = 0.0259 mmol/L, for triglycerides – 1 mg/dL = 0.0113 mmol/L

**S10 Table**. Unadjusted correlates of relative *CASR*, *RXRA*, *LXRA*, and *ENHO* transcript amounts among selected data of HD patients (n = 112)

| Parameter | Relative transcript amount | | | | | | | |
| --- | --- | --- | --- | --- | --- | --- | --- | --- |
|  | *CASR* (n = 112) | | *RXRA* (n = 111) | | *LXRA* (n = 111) | | *ENHO* (n = 112) | |
|  | β^a^ ± SE | P-value | β^a^ ± SE | P-value | β^a^ ± SE | P-value | β^a^ ± SE | P-value |
| Male gender | 0.214 ± 0.242 | 0.379 | 0.156 ± 0.090 | 0.085 | -0.029 ± 0.153 | 0.851 | 0.145 ± 0.107 | 0.178 |
| Age (per 10 years) | 0.006 ± 0.086 | 0.947 | -0.001 ± 0.032 | 0.965 | 0.098 ± 0.053 | 0.068 | -0.005 ± 0.038 | 0.898 |
| RRT duration (per 1 year) | **0.046 ± 0.023** | **0.042** | -0.014 ± 0.008 | 0.093 | 0.028 ± 0.014 | 0.052 | **-0.031 ± 0.010** | **0.002** |
| Diabetic nephropathy | 0.002 ± 0.305 | 0.994 | 0.065 ± 0.114 | 0.568 | -0.080 ± 0.191 | 0.675 | -0.051 ± 0.135 | 0.707 |
| Hypertensive/atherosclerotic nephropathy | -0.380 ± 0.303 | 0.213 | 0.066 ± 0.114 | 0.564 | -0.113 ± 0.191 | 0.554 | 0.097 ± 0.135 | 0.471 |
| Dry body weight (per 1 kg) | 0.018 ± 0.010 | 0.077 | **0.013 ± 0.003** | **0.0002** | **-0.020 ± 0.006** | **0.001** | 0.008 ± 0.004 | 0.054 |
| BMI (per 1 kg/m^2^) | 0.064 ± 0.035 | 0.072 | **0.045 ± 0.012** | **0.0003** | **-0.053 ± 0.021** | **0.016** | 0.018 ± 0.015 | 0.234 |
| Coronary artery disease | 0.241 ± 0.286 | 0.401 | 0.121 ± 0.107 | 0.260 | 0.326 ± 0.177 | 0.068 | -0.052 ± 0.127 | 0.683 |
| Antilipemic treatment | -0.320 ± 0.260 | 0.221 | 0.158 ± 0.097 | 0.107 | -0.058 ± 0.164 | 0.724 | -0.096 ± 0.115 | 0.405 |
| Active HBV/HCV infection | -0.038 ± 0.587 | 0.948 | 0.084 ± 0.219 | 0.703 | 0.156 ± 0.367 | 0.672 | -0.424 ± 0.256 | 0.101 |
| ALT (per 1 IU/L) | -0.014 ± 0.017 | 0.402 | 0.007 ± 0.006 | 0.274 | -0.0007 ± 0.010 | 0.947 | 0.007 ± 0.007 | 0.310 |
| AST (per 1 IU/L) | -0.029 ± 0.017 | 0.102 | 0.011 ± 0.007 | 0.083 | -0.009 ± 0.011 | 0.398 | 0.003 ± 0.008 | 0.681 |
| GGT (per 1 IU/L) | -0.0004 ± 0.002 | 0.823 | 0.001 ± 0.0007 | 0.131 | -0.000001 ± 0.001 | 0.999 | 0.00004 ± 0.0008 | 0.964 |
| ALP (per 1 IU/L) | -0.0008 ± 0.001 | 0.496 | 0.0008 ± 0.0004 | 0.071 | 0.0005 ± 0.0007 | 0.508 | 0.0004 ± 0.0005 | 0.458 |
| TG/HDL-cholesterol ratio ≥3.8 | 0.149 ± 0.242 | 0.540 | -0.081 ± 0.091 | 0.376 | -0.123 ± 0.152 | 0.418 | -0.081 ± 0.107 | 0.448 |
| Dyslipidemia by K/DOQI | 0.289 ± 0.243 | 0.236 | 0.051 ± 0.092 | 0.579 | 0.119 ± 0.153 | 0.438 | 0.078 ± 0.108 | 0.468 |
| LDL-cholesterol ≥ 100 mg/dL | 0.112 ± 0.242 | 0.645 | 0.103 ± 0.091 | 0.259 | 0.154 ± 0.152 | 0.311 | 0.094 ± 0.107 | 0.382 |
| Non-HDL-cholesterol ≥ 130 mg/dL and TG ≥ 200 mg/dL | 0.457 ± 0.320 | 0.156 | -0.013 ± 0.121 | 0.915 | 0.242 ± 0.201 | 0.230 | 0.011 ± 0.143 | 0.939 |
| C-reactive protein (per 1 mg/L) | -0.008 ± 0.011 | 0.481 | -0.001 ± 0.004 | 0.751 | 0.001 ± 0.007 | 0.831 | -0.002 ± 0.005 | 0.716 |
| Albumin (per 1 g/dL) | 0.379 ± 0.265 | 0.155 | 0.053 ± 0.100 | 0.598 | **-0.361 ± 0.165** | **0.030** | **0.294 ± 0.115** | **0.012** |
| Parathyroid hormone (per 100 pg/mL) | 0.027 ± 0.026 | 0.309 | 0.005 ± 0.010 | 0.606 | -0.011 ± 0.017 | 0.522 | -0.004 ± 0.012 | 0.760 |

Abbreviations: ALT – alanine aminotransferase, AST – aspartate aminotransferase, BMI – body mass index, GGT - ɣ-glutamyltransferase, RRT – renal replacement therapy

a – β coefﬁcient and SE values can be interpreted as follows: for a unitary change in an analyzed parameter, the relative transcript amount would change by β ± SE (ng/L)

**S11 Table**. Correlates of relative *CASR*, *RXRA*, *LXRA*, and *ENHO* transcript amounts among selected data of HD patients (n = 112) adjusted for dry body weight, RRT duration and serum albumin concentration, as appropriate

| Parameter | Relative transcript amount | | | | | | | |
| --- | --- | --- | --- | --- | --- | --- | --- | --- |
|  | *CASR* (n = 112) | | *RXRA* (n = 111) | | *LXRA* (n = 111) | | *ENHO* (n = 112) | |
|  | β^a^ ± SE | P-value | β^a^ ± SE | P-value | β^a^ ± SE | P-value | β^a^ ± SE | P-value |
| Male gender | 0.052 ± 0.257 | 0.838 | 0.022 ± 0.095 | 0.820 | 0.248 ± 0.155 | 0.113 | 0.031 ± 0.110 | 0.779 |
| Age (per 10 years) | 0.003 ± 0.009 | 0.684 | -0.00001 ± 0.003 | 0.997 | 0.008 ± 0.005 | 0.141 | 0.002 ± 0.037 | 0.613 |
| RRT duration (per 1 year) | **0.057 ± 0.022** | **0.013** | -0.009 ± 0.008 | 0.269 | 0.019 ± 0.013 | 0.173 | **-0.028 ± 0.010** | **0.005** |
| Diabetic nephropathy | 0.071 ± 0.304 | 0.816 | -0.009 ± 0.112 | 0.936 | -0.016 ± 0.185 | 0.930 | -0.090 ± 0.130 | 0.491 |
| Hypertensive/atherosclerotic nephropathy | -0.227 ± 0.298 | 0.448 | 0.074 ± 0.110 | 0.502 | -0.121 ± 0.181 | 0.506 | 0.061 ± 0.128 | 0.634 |
| Dry body weight (per 1 kg) | **0.019 ± 0.009** | **0.035** | **0.011 ± 0.003** | **0.0005** | **-0.016 ± 0.005** | **0.003** | 0.006 ± 0.004 | 0.144 |
| BMI (per 1 kg/m^2^) | 0.060 ± 0.075 | 0.426 | 0.025 ± 0.028 | 0.370 | 0.036 ± 0.046 | 0.440 | -0.045 ± 0.032 | 0.164 |
| Coronary artery disease | 0.321 ± 0.276 | 0.248 | 0.121 ± 0.101 | 0.236 | 0.326 ± 0.166 | 0.052 | -0.068 ± 0.119 | 0.567 |
| Antilipaemic treatment | -0.335 ± 0.250 | 0.183 | -0.156 ± 0.091 | 0.092 | -0.080 ± 0.154 | 0.603 | -0.072 ± 0.108 | 0.507 |
| Active HBV/HCV infection | -0.123 ± 0.768 | 0.873 | 0.308 ± 0.280 | 0.274 | -0.574 ± 0.464 | 0.218 | -0.142 ± 0.329 | 0.666 |
| ALT (per 1 IU/L) | -0.011 ± 0.017 | 0.477 | 0.006 ± 0.006 | 0.333 | 0.006 ± 0.010 | 0.577 | 0.0009 ± 0.007 | 0.902 |
| AST (per 1 IU/L) | -0.024 ± 0.018 | 0.181 | 0.007 ± 0.007 | 0.274 | 0.0013 ± 0.011 | 0.903 | -0.008 ± 0.008 | 0.312 |
| GGT (per 1 IU/L) | 0.0003 ± 0.002 | 0.870 | 0.0008 ± 0.0007 | 0.259 | 0.0009 ± 0.001 | 0.452 | -0.0008 ± 0.0008 | 0.309 |
| ALP (per 1 IU/L) | -0.0009 ± 0.001 | 0.457 | **0.0008 ± 0.0004** | **0.047** | 0.0002 ± 0.0007 | 0.786 | 0.0006 ± 0.0005 | 0.184 |
| TG/HDL-cholesterol ratio ≥3.8 | 0.023 ± 0.239 | 0.922 | -0.152 ± 0.087 | 0.083 | -0.008 ± 0.146 | 0.954 | -0.138 ± 0.102 | 0.178 |
| Dyslipidaemia by K/DOQI | 0.270 ± 0.258 | 0.297 | 0.062 ± 0.094 | 0.510 | 0.197 ± 0.153 | 0.200 | 0.149 ± 0.105 | 0.160 |
| LDL-cholesterol ≥ 100 mg/dL | -0.021 ± 0.237 | 0.928 | 0.093 ± 0.087 | 0.289 | 0.190 ± 0.144 | 0.188 | 0.106 ± 0.101 | 0.298 |
| Non-HDL-cholesterol ≥ 130 mg/dL and TG ≥ 200 mg/dL | 0.292 ± 0.239 | 0.223 | 0.093 ± 0.088 | 0.293 | 0.108 ± 0.146 | 0.463 | -0.019 ± 0.103 | 0.852 |
| C-reactive protein (per 1 mg/L) | -0.004 ± 0.011 | 0.732 | -0.002 ± 0.004 | 0.656 | -0.0004 ± 0.007 | 0.950 | 0.0002 ± 0.005 | 0.971 |
| Albumin (per 1 g/dL) | 0.372 ± 0.258 | 0.152 | 0.015 ± 0.100 | 0.879 | -0.302 ± 0.158 | 0.059 | **0.258 ± 0.110** | **0.021** |
| Parathyroid hormone (per 100 pg/mL) | 0.005 ± 0.027 | 0.851 | 0.009 ± 0.010 | 0.378 | -0.013 ± 0.016 | 0.428 | 0.0008 ± 0.012 | 0.944 |

Abbreviations: ALT – alanine aminotransferase, AST – aspartate aminotransferase, BMI – body mass index, GGT - ɣ-glutamyltransferase, RRT – renal replacement therapy

a – β coefﬁcient and SE values can be interpreted as follows: for a unitary change in an analyzed parameter, the relative transcript amount would change by β ± SE

**S12 Table.** *CASR*, ENHO, *RXRA*, and *LXRA* SNPs and relative amounts of their transcripts in all HD patients

| SNP | Relative transcript amount | | | Mode  of inheritance | P-value^a^ | Adjusted P-value^b^ |
| --- | --- | --- | --- | --- | --- | --- |
|  | Homozygosity for the major allele | Heterozygosity | Homozygosity for the variant allele |  |  |  |
| *CASR*  rs7652589  n = 112 | 0.359 (0.086 – 4.714), n = 35 | 0.313 (0.041 – 5.063), n = 54 | 0.436 (0.065 – 7.796), n = 23 | dominant | 0.903 | 0.204 |
|  |  |  |  | recessive | 0.372 | 0.079 |
|  |  |  |  | additive | 0.466 | 0.109 |
| *CASR*  rs1801725  n = 112 | 0.426 (0.041 – 5.063), n = 78 | 0.216 (0.065 – 7.796), n = 32 | 0.119 (0.062 – 0.177), n = 2 | dominant | 0.058 | 0.743 |
|  |  |  |  | recessive | 0.102 | 0.698 |
|  |  |  |  | additive | 0.093 | 0.565 |
| *RXRA*  rs10881578  n = 111 | 0.783 (0.113 – 2.228), n = 53 | 0.924 (0.095 – 2.813), n = 43 | 0.594 (0.109 – 1.984), n = 15 | dominant | 0.894 | 0.858 |
|  |  |  |  | recessive | 0.135 | 0.327 |
|  |  |  |  | additive | 0.193 | 0.691 |
| *RXRA*  rs10776909  n = 111 | 0.798 (0.109 – 2.813), n = 73 | 0.896 (0.095 – 1.984), n = 35 | 0.415 (0.316 – 0.613), n = 3 | dominant | 0.943 | 0.826 |
|  |  |  |  | recessive | 0.062 | 0.052 |
|  |  |  |  | additive | 0.051 | 0.051 |
| *RXRA*  rs749759  n = 111 | 0.794 (0.109 – 2.228), n = 50 | 0.863 (0.095 – 2.813), n = 55 | 0.624 (0.316 – 0.928), n = 6 | dominant | 0.597 | 0.908 |
|  |  |  |  | recessive | 0.161 | 0.182 |
|  |  |  |  | additive | 0.209 | 0.125 |
| *LXRA*  rs2279238  n = 111 | 1.056 (0.012 – 7.117), n = 78 | 0.812 (0.155 – 1.860), n = 33 | n = 0 | dominant | 0.198 | 0.175 |
|  |  |  |  | recessive | NA | NA |
|  |  |  |  | additive | NA | NA |
| *LXRA*  rs7120118  n = 111 | 1.086 (0.028 – 7.117), n = 53 | 0.962 (0.012 – 1.860), n = 49 | 0.561 (0.155 – 0.948), n = 9 | dominant | 0.058 | 0.050 |
|  |  |  |  | recessive | **0.002** | **0.042** |
|  |  |  |  | additive | **0.004** | 0.067 |
| *LXRA*  rs11039155  n = 111 | 1.055 (0.012 – 7.117), n = 79 | 0.870 (0.155 – 1.860), n = 32 | n = 0 | dominant | 0.284 | 0.213 |
|  |  |  |  | recessive | NA | NA |
|  |  |  |  | additive | NA | NA |
| ENHO  rs2281997  n = 112 | 0.714 (0.030 – 2.524), n = 58 | 0.614 (0.038 – 2.448), n = 50 | 0.463 (0.232 – 0.958), n = 4 | dominant | 0.434 | 0.290 |
|  |  |  |  | recessive | 0.481 | 0.787 |
|  |  |  |  | additive | 0.398 | 0.595 |
| ENHO  rs72735260  n = 112 | 0.658 (0.030 – 2.524), n = 81 | 0.789 (0.035 – 2.466), n = 31 | n = 0 | dominant | 0.691 | 0.536 |
|  |  |  |  | recessive | NA | NA |
|  |  |  |  | additive | NA | NA |

a - Mann Whitney test

b - adjustment for RRT duration, dry body mass and serum albumin concentration

Significant differences are indicated using bold font.

**S13 Table.** Correlations between *CASR*, *RXRA, LXRA*, and *ENHO* transcripts and TG/HDL-cholesterol ratio and serum lipid concentrations

1. all tested HD patients

| Pair of variables | \| N \| \| --- \| | \| Spearman’s rank-order correlation coefficient \| \| --- \| | \| t(N-2) \| \| --- \| | \| P-value \|  \| \| --- \| --- \| | Adjusted  P-value^a^ |
| --- | --- | --- | --- | --- | --- | --- | --- | --- | --- | --- |
| \| TG/HDL-cholesterol ratio & *CASR* \| \| --- \| | 112 | 0.158966 | 1.68872 | 0.094106 | 0.783 |
| \| TG/HDL-cholesterol ratio & *ENHO* \| \| --- \| | 112 | -0.057660 | -0.60575 | 0.545930 | 0.071 |
| \| TG/HDL-cholesterol ratio & *LXRA* \| \| --- \| | 111 | -0.072478 | -0.75869 | 0.449675 | 0.699 |
| \| TG/HDL-cholesterol ratio & *RXRA* \| \| --- \| | 111 | -0.008345 | -0.08713 | 0.930729 | 0.661 |
| \| Total Cholesterol & *CASR* \| \| --- \| | 112 | 0.108932 | 1.14933 | 0.252915 | 0.247 |
| \| Total Cholesterol & *ENHO* \| \| --- \| | 112 | 0.197359 | 2.11145 | 0.036999 | 0.083 |
| \| Total Cholesterol & *LXRA* \| \| --- \| | 111 | -0.052559 | -0.54949 | 0.583792 | 0.366 |
| \| Total Cholesterol & *RXRA* \| \| --- \| | 111 | 0.137517 | 1.44949 | 0.150071 | 0.249 |
| \| HDL-cholesterol & *CASR* \| \| --- \| | 112 | -0.129984 | -1.37494 | 0.171943 | 0.873 |
| \| HDL-cholesterol & *ENHO* \| \| --- \| | 112 | 0.102243 | 1.07798 | 0.283402 | 0.193 |
| \| HDL-cholesterol & *LXRA* \| \| --- \| | 111 | -0.011786 | -0.12306 | 0.902286 | 0.557 |
| \| HDL-cholesterol & *RXRA* \| \| --- \| | 111 | 0.034428 | 0.35965 | 0.719803 | 0.698 |
| \| LDL-cholesterol & *CASR* \| \| --- \| | 112 | 0.085817 | 0.90339 | 0.368292 | 0.330 |
| \| LDL-cholesterol & *ENHO* \| \| --- \| | 112 | 0.147437 | 1.56342 | 0.120827 | 0.023 |
| \| LDL-cholesterol & *LXRA* \| \| --- \| | 111 | -0.033761 | -0.35267 | 0.725014 | 0.806 |
| \| LDL-cholesterol & *RXRA* \| \| --- \| | 111 | 0.093239 | 0.97770 | 0.330386 | 0.539 |
| \| TG & *CASR* \| \| --- \| | 112 | 0.102441 | 1.08010 | 0.282461 | 0.540 |
| \| TG & *ENHO* \| \| --- \| | 112 | 0.007962 | 0.08351 | 0.933597 | 0.118 |
| \| TG & *LXRA* \| \| --- \| | 111 | -0.116394 | -1.22351 | 0.223776 | 0.901 |
| \| TG & *RXRA* \| \| --- \| | 111 | 0.024266 | 0.25342 | 0.800420 | 0.326 |
| \| Non-HDL-cholesterol & *CASR* \| \| --- \| | 112 | 0.145516 | 1.54261 | 0.125798 | 0.247 |
| \| Non-HDL-cholesterol & *ENHO* \| \| --- \| | 112 | 0.156438 | 1.66119 | 0.099523 | 0.154 |
| \| Non-HDL-cholesterol & *LXRA* \| \| --- \| | 111 | -0.040088 | -0.41887 | 0.676134 | 0.268 |
| \| Non-HDL-cholesterol & *RXRA* \| \| --- \| | 111 | 0.107470 | 1.12856 | 0.261563 | 0.276 |

B. HD patients not receiving lipid-lowering medication

| Pair of variables | \| N \| \| --- \| | \| Spearman’s rank-order correlation coefficient \| \| --- \| | \| t(N-2) \| \| --- \| | \| P-value \| \| --- \| | Adjusted  P-value^a^ |
| --- | --- | --- | --- | --- | --- | --- | --- | --- | --- |
| \| TG/HDL-cholesterol ratio & *CASR* \| \| --- \| | 77 | 0.139668 | 1.22153 | 0.225711 | 0.940 |
| \| TG/HDL-cholesterol ratio & *ENHO* \| \| --- \| | 77 | -0.140522 | -1.22915 | 0.222857 | 0.083 |
| \| TG/HDL-cholesterol ratio & *LXRA* \| \| --- \| | 76 | -0.093044 | -0.80388 | 0.424039 | 0.863 |
| \| TG/HDL-cholesterol ratio & *RXRA* \| \| --- \| | 76 | 0.008121 | 0.06986 | 0.944495 | 0.798 |
| \| Total Cholesterol & *CASR* \| \| --- \| | 77 | 0.124544 | 1.08705 | 0.280498 | 0.257 |
| \| Total Cholesterol & *ENHO* \| \| --- \| | 77 | 0.044740 | 0.38785 | 0.699226 | 0.825 |
| \| Total Cholesterol & *LXRA* \| \| --- \| | 76 | 0.073534 | 0.63428 | 0.527852 | 0.259 |
| \| Total Cholesterol & *RXRA* \| \| --- \| | 76 | 0.067491 | 0.58190 | 0.562400 | 0.416 |
| \| HDL-cholesterol & *CASR* \| \| --- \| | 77 | -0.122375 | -1.06782 | 0.289025 | 0.663 |
| \| HDL-cholesterol & *ENHO* \| \| --- \| | 77 | 0.063325 | 0.54951 | 0.584285 | 0.997 |
| \| HDL-cholesterol & *LXRA* \| \| --- \| | 76 | 0.059210 | 0.51024 | 0.611404 | 0.638 |
| \| HDL-cholesterol & *RXRA* \| \| --- \| | 76 | -0.006238 | -0.05367 | 0.957346 | 0.702 |
| \| LDL-cholesterol & *CASR* \| \| --- \| | 77 | 0.107497 | 0.93638 | 0.352083 | 0.289 |
| \| LDL-cholesterol & *ENHO* \| \| --- \| | 77 | 0.023006 | 0.19929 | 0.842575 | 0.099 |
| \| LDL-cholesterol & *LXRA* \| \| --- \| | 76 | 0.061456 | 0.52967 | 0.597928 | 0.638 |
| \| LDL-cholesterol & *RXRA* \| \| --- \| | 76 | 0.002967 | 0.02552 | 0.979707 | 0.901 |
| \| TG & *CASR* \| \| --- \| | 77 | 0.078759 | 0.68420 | 0.495960 | 0.658 |
| \| TG & *ENHO* \| \| --- \| | 77 | -0.150707 | -1.32024 | 0.190771 | 0.028 |
| \| TG & *LXRA* \| \| --- \| | 76 | -0.098743 | -0.85359 | 0.396088 | 0.932 |
| \| TG & *RXRA* \| \| --- \| | 76 | -0.005702 | -0.04905 | 0.961009 | 0.529 |
| \| Non-HDL-cholesterol & *CASR* \| \| --- \| | 77 | 0.163997 | 1.43975 | 0.154100 | 0.298 |
| \| Non-HDL-cholesterol & *ENHO* \| \| --- \| | 77 | -0.000394 | -0.00342 | 0.997284 | 0.820 |
| \| Non-HDL-cholesterol & *LXRA* \| \| --- \| | 76 | 0.066669 | 0.57479 | 0.567176 | 0.223 |
| \| Non-HDL-cholesterol & *RXRA* \| \| --- \| | 76 | 0.047432 | 0.40848 | 0.684100 | 0.467 |

C. HD patients receiving lipid-lowering medication

| Pair of variables | \| N \| \| --- \| | \| Spearman’s rank-order correlation coefficient \| \| --- \| | \| t(N-2) \| \| --- \| | \| P-value \|  \| \| --- \| --- \| | Adjusted  P-value^a^ |
| --- | --- | --- | --- | --- | --- | --- | --- | --- | --- | --- |
| \| TG/HDL-cholesterol ratio & *CASR* \| \| --- \| | 35 | 0.164986 | 0.96094 | 0.343570 | 0.734 |
| \| TG/HDL-cholesterol ratio & *ENHO* \| \| --- \| | 35 | 0.103922 | 0.60023 | 0.552450 | 0.320 |
| \| TG/HDL-cholesterol ratio & *LXRA* \| \| --- \| | 35 | -0.063025 | -0.36277 | 0.719087 | 0.779 |
| \| TG/HDL-cholesterol ratio & *RXRA* \| \| --- \| | 35 | -0.016246 | -0.09334 | 0.926197 | 0.419 |
| \| Total Cholesterol & *CASR* \| \| --- \| | 35 | 0.036292 | 0.20862 | 0.836027 | 0.535 |
| \| Total Cholesterol & *ENHO* \| \| --- \| | 35 | 0.497863 | 3.29777 | 0.002340 | 0.044 |
| \| Total Cholesterol & *LXRA* \| \| --- \| | 35 | -0.286135 | -1.71544 | 0.095641 | 0.556 |
| \| Total Cholesterol & *RXRA* \| \| --- \| | 35 | 0.256568 | 1.52492 | 0.136807 | 0.304 |
| \| HDL-cholesterol & *CASR* \| \| --- \| | 35 | -0.179385 | -1.04748 | 0.302491 | 0.499 |
| \| HDL-cholesterol & *ENHO* \| \| --- \| | 35 | 0.209936 | 1.23348 | 0.226111 | 0.032 |
| \| HDL-cholesterol & *LXRA* \| \| --- \| | 35 | -0.150375 | -0.87377 | 0.388556 | 0.406 |
| \| HDL-cholesterol & *RXRA* \| \| --- \| | 35 | 0.093897 | 0.54179 | 0.591603 | 0.989 |
| \| LDL-cholesterol & *CASR* \| \| --- \| | 35 | 0.049170 | 0.28280 | 0.779095 | 0.577 |
| \| LDL-cholesterol & *ENHO* \| \| --- \| | 35 | 0.401205 | 2.51613 | 0.016909 | 0.156 |
| \| LDL-cholesterol & *LXRA* \| \| --- \| | 35 | -0.181411 | -1.05971 | 0.296972 | 0.169 |
| \| LDL-cholesterol & *RXRA* \| \| --- \| | 35 | 0.298942 | 1.79959 | 0.081076 | 0.326 |
| \| TG & *CASR* \| \| --- \| | 35 | 0.115554 | 0.66829 | 0.508599 | 0.466 |
| \| TG & *ENHO* \| \| --- \| | 35 | 0.278451 | 1.66545 | 0.105291 | 0.972 |
| \| TG & *LXRA* \| \| --- \| | 35 | -0.211920 | -1.24568 | 0.221655 | 0.914 |
| \| TG & *RXRA* \| \| --- \| | 35 | 0.046642 | 0.26823 | 0.790192 | 0.556 |
| \| Non-HDL-cholesterol & *CASR* \| \| --- \| | 35 | 0.115843 | 0.66998 | 0.507535 | 0.415 |
| \| Non-HDL-cholesterol & *ENHO* \| \| --- \| | 35 | 0.451884 | 2.90992 | 0.006428 | 0.114 |
| \| Non-HDL-cholesterol & *LXRA* \| \| --- \| | 35 | -0.243031 | -1.43926 | 0.159496 | 0.676 |
| \| Non-HDL-cholesterol & *RXRA* \| \| --- \| | 35 | 0.238129 | 1.40846 | 0.168347 | 0.278 |

a – adjustment for RRT duration, dry body mass and serum albumin concentration

**Supplementary figures**

**S1 Fig. Chromosomal localization of *CASR*, the Linkage Disequilibrium (LD) data between the tested *CASR* variants, and haplotypes formed by the examined *CASR* variants**

A. Chromosomal localization of *CASR*

*CASR* (ENST00000639785.1) is located on chromosome 3q13.33 – q21.1 and consists of 7 exons. Nucleotide variants tested in the present study are marked with arrows.

B. The Linkage Disequilibrium (LD) data between the tested *CASR* variants

In the D’ and r^2^ value plots, numbers denote D’ and r^2^ value expressed as a percentage of maximal value (1.00).

C. Haplotypes formed by the tested *CASR* variants

Both tested biallelic *CASR* variants have three possible genotypes (rs7652589: AA, AG, and GG; rs1801725: TT, GT, and GG). These genotypes form four haplotypes: GG, AG, GT, and AT.

**
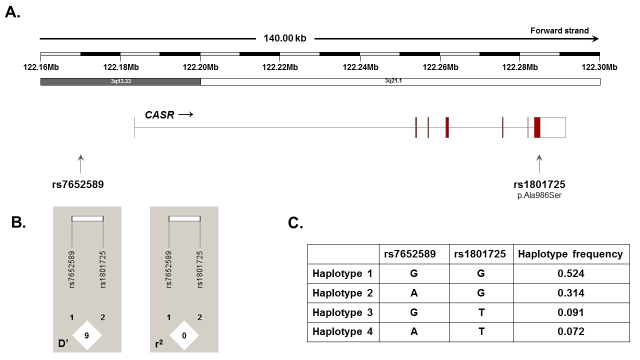
**

**S2 Fig. Correlations between serum LDL-cholesterol and non-HDL-cholesterol concerning serum TG levels in HD patients not receiving lipid-lowering medication**

1. TG < 150 mg/dL
2. TG ≥150 – 250 mg/dL

C. TG >250 mg/dL
